# Interhemispheric Integration after Callosotomy: A Meta-Analysis of Poffenberger and Redundant-Target Paradigms

**DOI:** 10.1007/s11065-022-09569-w

**Published:** 2022-12-09

**Authors:** René Westerhausen

**Affiliations:** Section for Cognitive and Clinical Neuroscience, Department of Psychology, University of Oslo, POB 1094 Blindern, Oslo, 0317 Norway

**Keywords:** Corpus callosum, Callosotomy, Commissurotomy, Split-brain, Brain asymmetry

## Abstract

The central role of the corpus callosum in integrating perception and cognition across the cerebral hemispheres makes it highly desirable for clinical and basic research to have a repertoire of experimental paradigms assessing callosal functioning. Here, the objective was to assess the validity of two such paradigms (Poffenberger, redundant-target paradigms) by conducting single-step meta-analyses on individual case data of callosotomy patients. Studies were identified by systematic literature search (source: Pubmed and WebOfKnowledge, date: 07.03.2022) and all studies were included that reported callosotomy case data for either paradigm. Twenty-two studies (38 unique cases) provided 116 observations of the crossed-uncrossed difference (CUD) for the Poffenberger paradigm, while ten studies (22 cases, 103 observations) provided bilateral redundancy gain (bRG) measures. Using linear-mixed models with “individual” and “experiment” as random-effects variable, the mean CUD was estimated at 60.6 ms (*CI*_*95%*_: 45.3; 75.9) for commissurotomy, 43.5 ms (26.7; 60.2) for complete callosotomy, and 8.8 ms (1.1; 16.6) for partial anterior-medial callosotomy patients. The estimates of commissurotomy/callosotomy patients differed significantly from patients with partial callosotomy and healthy controls. The mean bRG_min_ (minimum unilateral reference) was estimated at 42.8 ms (27.1;58.4) for patients with complete and 30.8 ms (16.8; 44.7) for patients with partial callosotomy, both differing significantly from controls. One limitation was that different formulas for bRG were used, making it necessary to split the sample and reducing test power of some analyses. Nevertheless, the present findings suggest that both paradigms assess interhemispheric callosal integration, confirming their construct validity, but likely test distinct callosal functions.

**Supplementary Information:**

The online version contains supplementary material available at 10.1007/s11065-022-09569-w.

## Introduction

The axons forming the corpus callosum represent the major connection between the cerebral hemispheres and have an important role in human perception and cognition (Banich, 1998; Innocenti et al., 2022). Predominately contralateral ascending sensory pathways result in an interhemispheric separation of perceptual information which is unified via callosal axons (e.g., Genç et al., 2011; Steinmann et al., 2018; Westerhausen et al., 2009). Likewise, hemispheric specialization for various cognitive functions (e.g., left-hemispheric dominance for language processing) demands both integration of information and coordination of processing between the hemispheres to allow for efficient cognitive processing (e.g., Chechlacz et al., 2015; Labache et al., 2020; Thiel et al., 2006). Corpus callosum functioning undergoes systematic changes in early development or in older age (e.g., Meissner et al., 2017; Scally et al., 2018; van der Cruyssen et al., 2020; Westerhausen et al., 2015) and pathological alterations in callosal interaction are linked to neurological and psychiatric conditions (e.g., Lodhia et al., 2017; Steinmann et al., 2017; Warlop et al., 2008; Whitford et al., 2011). Thus, experimental paradigms for the assessment of interhemispheric interaction via the corpus callosum are highly relevant to gain a better understanding of the neuronal foundations of healthy and pathological cognition.

The present meta-analysis examines the validity of two classes of paradigms thought to assesses interhemispheric interaction via the corpus callosum, namely *Poffenberger paradigms* (e.g., Bashore, 1981; Braun, 1992; Marzi, 1999; Poffenberger, 1912) and the *bilateral redundant-target paradigms* (e.g., Corballis, 2002; Reuter-Lorenz et al., 1995). This is achieved by integrating published data from so-called “split-brain” patients. In these patients, the surgical transection of the corpus callosum – alone (i.e., callosotomy) or together with the other commissural tracts (i.e., commissurotomy) – severely impedes or prevents any direct interaction between the cerebral hemispheres (Gazzaniga, 2000; Lassonde & Ouimet, 2010; Volz & Gazzaniga, 2017), which should substantially affect performance in paradigms attempting to assess this interaction. Thus, studies on these patients are a critical test for the validity of paradigms testing hemispheric interaction. However, one major drawback with studies on split-brain patients is that they are case studies and include at best a small series of patients. This limits the generalizability of the findings and potentially encourages overinterpretation (Nissen & Wynn, 2014) since case studies cannot distinguish case-specific from general brain characteristics. Idiosyncratic aspects of the underlying brain pathology that indicated the surgery or specific features/adverse effects of the surgery itself (e.g., extend of the callosal surgery or possible additional brain damage) might have caused the phenomenon in question and not the fact that the callosal axons have been transected. One approach, which can help overcoming the ambiguity around the split-brain literature, obviously is the integration of evidence across multiple cases. This can be accomplished by narrative review or, preferably, by meta-analytic statistical integration of the published data. While the classical effect-size based meta-analyses are difficult to conduct on case data, publications on split-brain patients usually report outcome variables in very detailed fashion making it possible to conduct meta-analyses based on individual participant data (Burke et al., 2017). For example, applying this method, a recent meta-analysis indicated that callosotomy has a negative effect on the patients’ performance IQ in patients which show average performance levels before the surgery (Westerhausen & Karud, 2018).

**Fig. 1:**
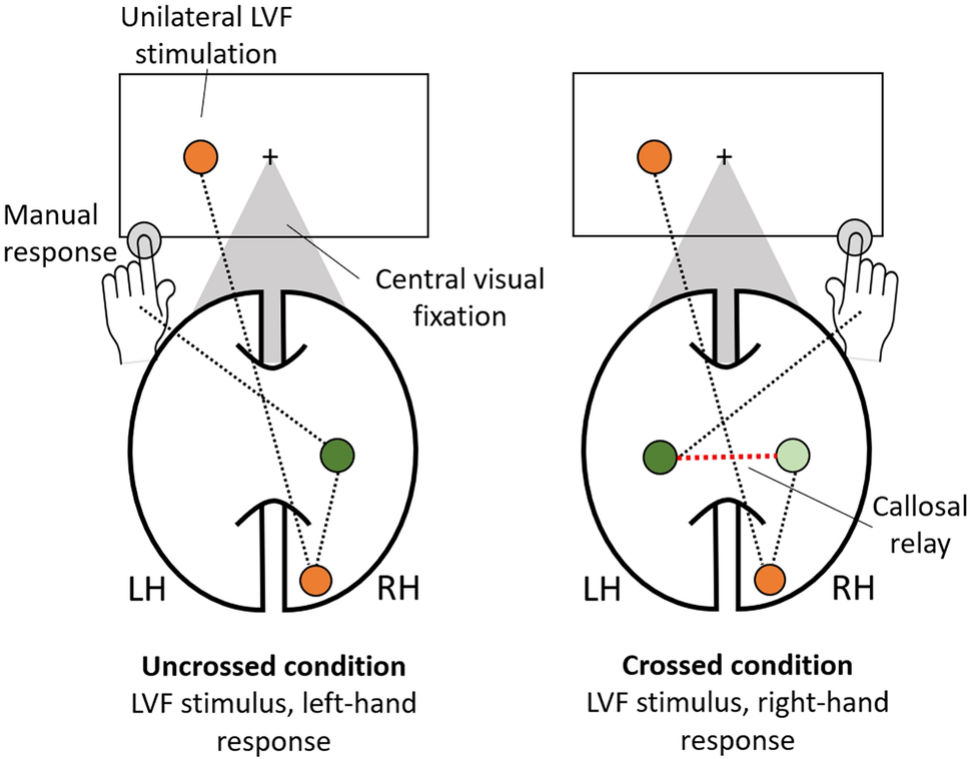
Illustration of the Poffenberger paradigm, exemplified for stimulation of the left visual field (LVF). The LVF stimulation leads to an initial contralateral representation of the stimulus in the right hemisphere (RH). According to the callosal-relay model, responding with the left hand to LVF stimulation, relies solely on *intra*hemispheric transfer of information as the left hand is also controlled by the RH. However, in the “crossed condition” (i.e., LVF stimulation and right-hand response) an additional *inter*hemispheric transfer via the corpus callosum is required. The additional callosal transfer step is thought to result in response-time difference between crossed and uncrossed conditions (CUD). Callosotomy prevents the callosal transfer in the crossed condition, further increasing the CUD, as alternative (subcortical) routes for the interhemispheric information transfer have to be utilized

The Poffenberger and bilateral redundant-target paradigms are closely related, as both rely on the brief para-foveal stimulation with basic visual stimuli (e.g., light flashes or simple shapes) and require fast simple (no choice alternatives) manual responses (Corballis, 2002; Reuter-Lorenz et al., 1995). Poffenberger paradigms confront the participants with repeated unilateral stimulus presentations either to the left or right peripheral visual field, with the instruction to respond as quickly as possible to the appearance of the stimulus. The responses are given either with the left or right hand (e.g., by button press) while the responding hand is varied within the experiment. As a result, as illustrated in Fig. 1, responses are either given from the same hemisphere that was stimulated (called uncrossed condition; e.g., right-hand response after stimulating the right visual field) or from the opposite hemisphere (crossed condition; e.g., right response after left stimulation). Typically, in healthy participants, the average response time of the crossed condition is 2 to 4 ms slower than of the uncrossed condition (for meta-analyses see Braun, 1992; Marzi et al., 1991; for more recent studies see e.g., Friedrich et al., 2017; Scally et al., 2018; Westerhausen et al., 2006). This so-called crossed-uncrossed difference (CUD) is traditionally interpreted as the time it takes to “relay” the information present in one hemisphere to the opposite hemisphere to initiate the manual response (Marzi, 1999; Marzi et al., 1991; Poffenberger, 1912). This interpretation assumes that both hemispheres are equally competent in perceiving the stimulus and initiating the response, so that difference between contralateral and ipsilateral response can be attributed solely to the interhemispheric transfer (Braun et al., 1994). Alternative accounts interpret the CUD as the time it takes one hemisphere to co-activate with the opposite hemisphere for response initiation (Kinsbourne, 2003; Miller, 2004).

Importantly, both callosal transfer and co-activation models predict that sectioning of the corpus callosum leads to delay of the crossed response and, consequently, an increased CUD, as the interaction between the hemispheres has to be routed via other (presumably) subcortical commissures (Corballis et al., 2018; Savazzi et al., 2007). In line with this prediction, the literature appears somewhat consistent in that complete section of the corpus callosum leads to an increase in the CUD compared to controls (e.g., Aglioti et al., 1993; Clarke & Zaidel, 1989; Corballis, 1998; Corballis et al., 2003; Iacoboni et al., 2000; Ouimet et al., 2010; Savazzi et al., 2007; Sergent & Myers, 1985). However, it is less clear whether the CUD is significantly increased compared to controls after partial callosotomy (Corballis et al., 2003; Di Stefano et al., 1992; Iacoboni & Zaidel, 1995; Ouimet et al., 2010), or which location of partial callosotomy (e.g., anterior vs. posterior) might be required to cause the CUD increase (Corballis et al., 2003; Jeeves et al., 2001; Savazzi et al., 2007). Differential findings, depending on which callosal region is sectioned, might inform about whether the hemispheric integration takes place on sensory, motor, or higher cognitive level. It also remains to be established whether there is a difference between commissurotomy and pure callosotomy patients in the magnitude of the effect. One might predict that the sectioning of anterior and other forebrain commissures in addition to the corpus callosum might further prolong the delay in hemispheric interaction after commissurotomy compared to callosotomy patients.

Redundant-target paradigms elicit the general phenomenon that response times are faster when two (redundant) target stimuli are presented than when a single target is presented (e.g., Gondan & Minakata, 2016; Hershenson, 1962; Miller, 1982; Schmid & Schenk, 2022), a phenomenon referred to as *redundancy gain*. The relevant implementations of the paradigm represent a special case since the redundant target stimuli are presented in opposite visual fields, thereby providing bilateral redundancy (Corballis, 2002; Miniussi et al., 1998; Reuter-Lorenz et al., 1995; Schulte et al., 2004). That is, one stimulus is presented to the left and the other, simultaneously, to the right hemi-field while the participant are asked to press a button as quickly as possible. The bilateral redundancy gain (bRG) is then determined in comparison to the response time of this bilateral condition to a unilateral stimulus condition. Thus, in its most simple form, bRG paradigms are an extension of Poffenberger paradigms by introducing a bilateral in addition to the unilateral conditions. The bRG varies depending on the paradigm set-up and the exact method it is calculated, but for simple bilateral paradigms, it is usually in the range of 12 to 30 ms in healthy young individuals (e.g., Corballis, 2002; Linnet & Roser, 2012; Ouimet et al., 2009; Savazzi & Marzi, 2008; Schulte et al., 2004).

While various models have addressed the general phenomenon of the redundant-target effect (for review see Gondan & Minakata, 2016; Miller, 1982, 2004), the bilateral condition has received special attention as studies on split-brain patients produced a surprising outcome (Corballis, 1998; Miller, 2004; Reuter-Lorenz et al., 1995). While intuitively one would predict that sectioning of the corpus callosum reduces the integration of information across the visual half fields and, in turn, reduces (if not eliminates) the bRG, studies consistently report an enhanced bRG in patients compared to controls (Corballis, 1998; Corballis et al., 2003, 2005; Iacoboni et al., 2000; Ouimet et al., 2009; Pollmann & Zaidel, 1999; Reuter-Lorenz et al., 1995; Roser & Corballis, 2002, 2003; Savazzi & Marzi, 2004). Attempting to explain this counterintuitive increase in bRG after callosotomy, two models emerged both claiming that enhancement is not driven by an increased response-time gain in the bilateral condition but rather reflects an increased response time in the unilateral reference condition (Miller, 2004; Reuter-Lorenz et al., 1995). That is, both hemispheres need to be coactivated to initiate a fast response (“AND” gate), and that also the unilateral condition involves a process of callosal integration. For example, Miller (2004) in his (graded) hemispheric co-activation model suggests that coactivation of the two hemispheres can be achieved either via bilateral stimulation or indirectly via the corpus callosum after unilateral stimulation. Thus, the model states that even if stimulus reception and response are handled by the same hemisphere (as in the uncrossed condition of the Poffenberger paradigms), a callosal integration step is required. Miller justifies this claim by referring to the observation of ipsi- in addition to contralateral cortical activation during unilateral motor tasks (e.g., Bernard et al., 2002; Ikeda et al., 1995). Sectioning of callosal connections, consequently, results in a delay of the co-activation and response after unilateral stimulation, as slower subcortical pathways have to be used. In the bilateral condition, no response delay should be observed since both hemispheres are engaged/activated via direct stimulation. Miller (2004) also claims that the split-brain literature supports this notion by showing that the response time is unaltered after bilateral stimulation and increased after unilateral stimulation. An inspection of the available case studies (listed above) does not readily confirm this statement, warranting statistical testing of this hypothesis in the present meta-analysis. Additionally, while the literature in general appears consistent in finding the enhanced bRG after split-brain surgery, it is less obvious from the available literature whether commissurotomy, complete callosotomy, and partial callosotomy patients differ in the magnitude of the bRG, or whether it only occurs when specific callosal subsections were severed.

Finally, CUD and bRG can be tested within the same experimental paradigm (e.g., Corballis, 2002; Ouimet et al., 2009) and are theoretically related (Miller, 2004) so that appears reasonable to ask how these two indices are correlated, although predictions in opposing directions can be made (Corballis, 2002). If both CUD and bRG rely on the same callosal processing steps, a prolonged CUD should negatively affect the redundancy gain as the neuronal integration between the hemispheres would be slower. That is, a negative correlation of CUD and bRG would be predicted. However, the findings that split-brain patients show an enlarged CUD and enhanced bRG, a positive association might also be predicted. Within Miller’s model (Miller, 2004), a smaller as compared to a larger CUD reflects a faster coactivation between the hemispheres in the unilateral condition, which in turn, should also reduce the bRG. Corballis (2002) tested the association of both indices in a sample of 58 healthy individuals and did not find a significant correlation (all *|r|*< 0.14). This finding suggests partial independence of the two phenomena, and Corballis (2002) speculates that different callosal systems and subregions might serve the two functions. Nevertheless, the association of CUD and bRG might be accentuated in split-brain patients since surgery results in stronger inter-individual variations and potentially stronger effect sizes.

Following the discussion above, the overall aim of the present study was to collect, integrate, and re-analyze all individual data of split-brain patients available in the literature representing measures of CUD and bRG, respectively. Relevant studies were identified and analyzed using a systematic literature search adhering to PRISMA criteria (Page et al., 2021). The retrieved individual participant data of both paradigms were subject to a series of one-step meta-analyses to test for (a) the statistical significance of CUD and bRG in the patient sample, (b) the difference of patients from healthy controls in CUD and bRG, as well as (c) CUD/bRG differences between patients with commissurotomy, complete callosotomy, and partial callosotomy. Furthermore, because of their theoretical significance (d) the mean response time measures for the uncrossed unilateral condition of the Poffenberger paradigm and the bilateral condition of bilateral redundant paradigms were tested for differences between patients and controls. Finally, (e) the association of CUD and bRG in patient was tested to evaluate whether both phenomena rely on separate or related callosal integration mechanisms. All analyses were conducted using linear-mixed modelling to statistically account for differences between individual patients and experimental set-ups (both included as random effects) when evaluating the effects of interest.

## Material and Methods

### Literature Search and Study Identification

The systematic literature search was conducted on 07.03.2022 in Pubmed and Web of Knowledge (core collection). No additional search in conference proceedings was conducted (beyond the conference abstracts available in these databases) as more contemporary studies cannot be expected given the split-brain procedure has gone out of fashion. The search query used for both databases included a search-term list to select studies examining split-brain patients combined with task-specific terms. That is, the search was combination of the query ("split-brain" OR "callosotomy" OR "commissurotomy") with ("interhemispheric transfer" OR "inter-hemispheric transfer" OR "Poffenberger" OR “crossed-uncrossed”) and (“redundant target” OR “redundancy signal” OR “redundancy gain”), respectively. The search-term list for patients was combined with each task-specific list with an “AND”. The search was conducted in “All Fields” and without any restriction of the time period.

Regarding the Poffenberger paradigm, the search in Pubmed yielded 82, the search in Web of Knowledge 155 records. Regarding the redundant-target paradigm, the search in Pubmed found 12 records, the search in Web of Knowledge 42 records. The results for both paradigms were exported to separate Endnote libraries and duplicates were removed. This left 183 and 42 unique records of the Poffenberger and redundant-target paradigm search, to which 4 and 1 studies, respectively, were added that were identified via references. The resulting 187 and 43 studies were screened for relevance using a checklist containing four criterion questions: (a) Is it an empirical study?; (b) Does the study examine a human sample?; (c) Does the study report results from the relevant paradigm (Poffenberger/redundant-target paradigm)?; and (d) Were patient examined who had undergone commissurotomy (brain) or complete/partial callosotomy? These questions were answered by screening title and abstracts or, where necessary, by consulting the full article. The questions were addressed successively and a negative answer to one the questions led to the exclusion of the article without considering the remaining screening questions.

**Fig. 2:**
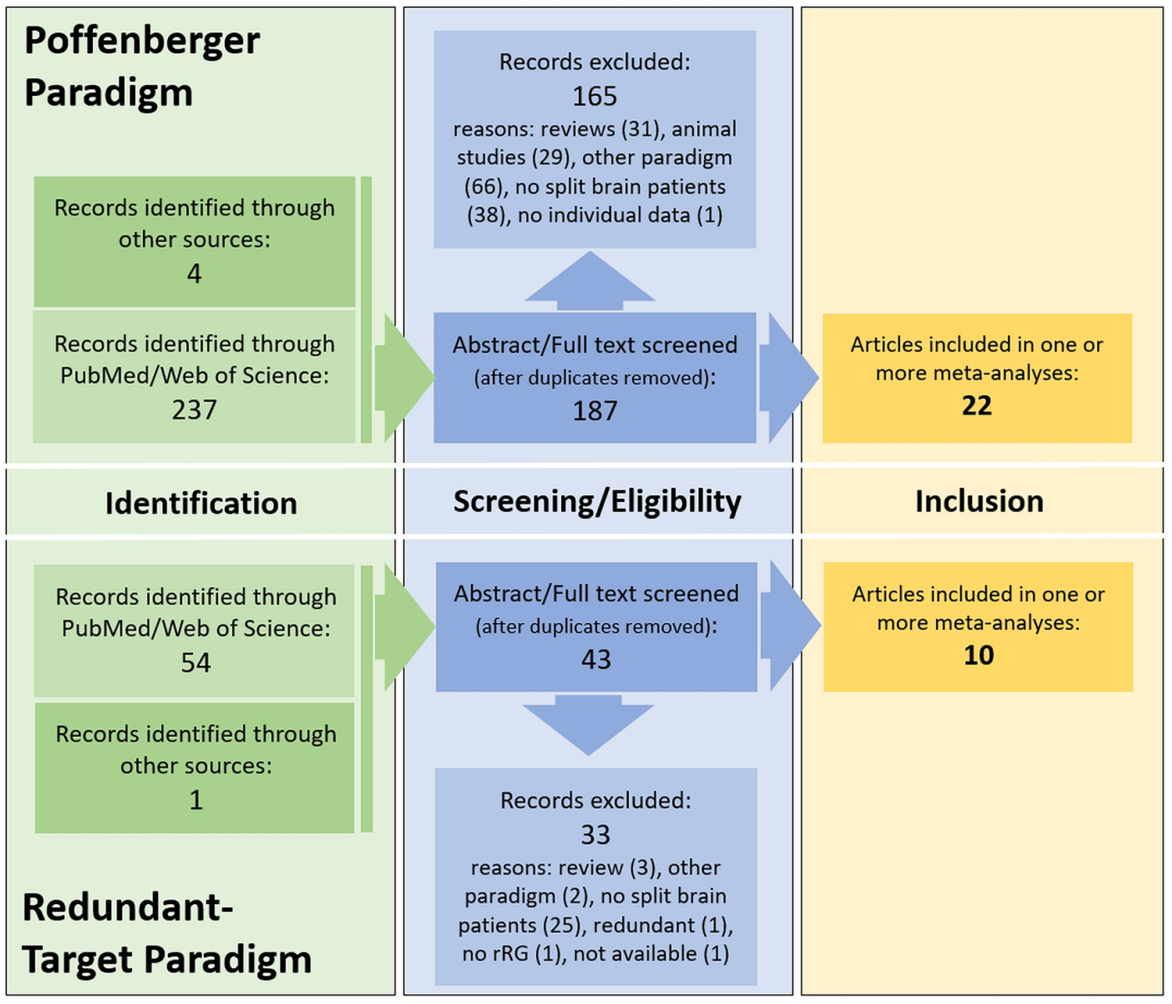
Flow-chart summarizing the inclusion of studies into the present meta-analysis. Separately for Poffenberger (top half) and redundant-target paradigm (bottom half) the numbers of considered studies after initial identification (database search) as well as following the screening of abstracts and study text is reported. The number of studies included in the meta-analysis is also provided

As shown in Fig. 2, of the above identified 187 Poffenberger articles, 31 were excluded since they represented reviews, 29 were excluded since animal research was reported, 66 were excluded as no Poffenberger paradigm was used, and 38 were excluded as split-brain patients were not tested. In addition, one study was not suited for the meta-analysis as individual data was not presented (Smith, 1947). Thus, a total of *k*_*s*_ = 22 studies contributed data to the meta-analyses regarding the CUD data.

Of the reviewed 43 studies identified by the redundant-target search, three were excluded as they were reviews, two did not use the paradigm, and 25 did not include a split-brain patient group. Of the remaining 12, two were excluded either as the data did not allow to calculate bRG (Corballis, Hamm, Barnett, & Corballis, 2002) or since it was an abstract of a published article that is included in the meta-analysis (Ouimet et al., 2008). For one identified publication data could not be retrieved even after contacting the authors (Marzi et al., 1997, cited from Pollmann & Zaidel, 1999). Thus, data from *k*_*s*_ = 10 studies was included in the present meta-analyses of bRG effects. Table 1 provides an overview of all included studies for the analyses of CUD and bRG. As can be seen, several studies provided data of both paradigms.

**Table 1:** Overview of included studies and patients per paradigm

		Complete Callosotomy		Partial Callosotomy		Controls	Paradigm		
#	Study	N	Cases^a^	N	Cases		CUD^b^	bRG	bRG reference^c^
1	Aglioti et al., 1993	1	ME	0		Yes (na)	❺		
2	Aglioti et al., 1996	1	ME	1	ID	Yes	❶		
3	Clarke & Zaidel, 1989	4	AA*, LB*, NG*, RY*	0		Yes	❹		
4	Corballis, 1998	3	JW, LB*, ME	0		Yes	❷	❷	avg, *min*, ips
5	Corballis et al., 2002	2	JW, ME	0		Yes	❶		
6	Corballis et al., 2003	1	DDV	3	LP, MC, RV	Yes	❶	❶	avg, min, ips
7	Corballis et al., 2005	2	DDV, JW	0		No	❶	❷	avg, min, ips
8	DiStefano et al., 1992	0		1	MP	Yes	❶		
9	Forster & Corballis, 1998	1	LB*	0		Yes	❷		
10	Iacoboni & Zaidel, 1995	2	LB*, NG*	1	DW	Yes	❸		
11	Iacoboni et al., 2000	4	DT, GC, LB*, NG*	3	BM, DW, JP	No	❶	❶	*ips*
12	Jeeves et al., 2001	0		2	SA, WS	Yes	❶		
13	Marzi et al., 1999	1	ME	0		Yes	❶		
14	McKeever et al., 1997	2	CAK, POV	1	MUR	No	❶		
15	Mooshagian et al., 2009	1	AA*	0		Yes	❶		
16	Ouimet et al., 2009	4	DDV, FB, ML, IC/YC^d^	4	AP, GS, MM, PM	Yes		❸	*min*
17	Ouimet et al., 2010	4	DDV, FB, ML, IC/YC	4	AP, GS, MM, PM	Yes	❷		
18	Pollmann & Zaidel, 1999	2	LB*, NG*	0		Yes		❷	avg, min, ips
19	Reuter-Lorenz et al, 1995	1	JW	1	SC^e^	Yes	❸	❹	*avg*, min, ips
20	Roser & Corballis, 2002	4	AA*, NG*, JW, VP	0		Yes	❶	❶	*avg*
21	Roser & Corballis, 2003	4	AA*, NG*, JW, VP	1		Yes	❷	❷	*min*
22	Savazzi & Marzi, 2004	1	DDV	0		Yes		❻	*avg*
23	Savazzi et al., 2007	2	FB, DDC	3	AZ, MB, MC	Yes	❸		
24	Sergent & Myers, 1985	2	LB*, NG*	0		Yes	❷		
25	Tassinari et al., 1994	1	(ME)^f^	7	BS, CG, FB, GE, GV, ID, VG	No	❶		

^a^Case names are given using the initials used in the literature, whereby * indicates a patient with commissurotomy (as opposed to callosotomy)

^b^numbers in circle indicate the number of experiments/conditions per case

^c^measure used as unilateral reference when determining bRG: *avg* average, *min* minimum, *ips* ipsilateral response time, *na* not available. Italicised text indicates the value provided in the original publication, while all others are calculated based on the available data

^d^IC/YC used in #16/#17 refer to same individual

^e^for SC only bRG data available

^f^same data as reported in #1

The meta-analysis was not preregistered and no explicit protocol was formulated, as it at the beginning of the project was not foreseeable which dependent variables (e.g., aggregate, as CUD, or per condition) would be available for a sufficient amount of cases to allow a meta-analysis.

### Data Extraction: Case Identification

Several patients were tested with various implementations of the Poffenberger/redundant-target paradigms so that data of the same cases appeared in more than one publication (see Table 1). Thus, special care was taken to identify and track patients by comparing the case names (i.e., case initials) and case descriptions across studies.

**Fig. 3:**
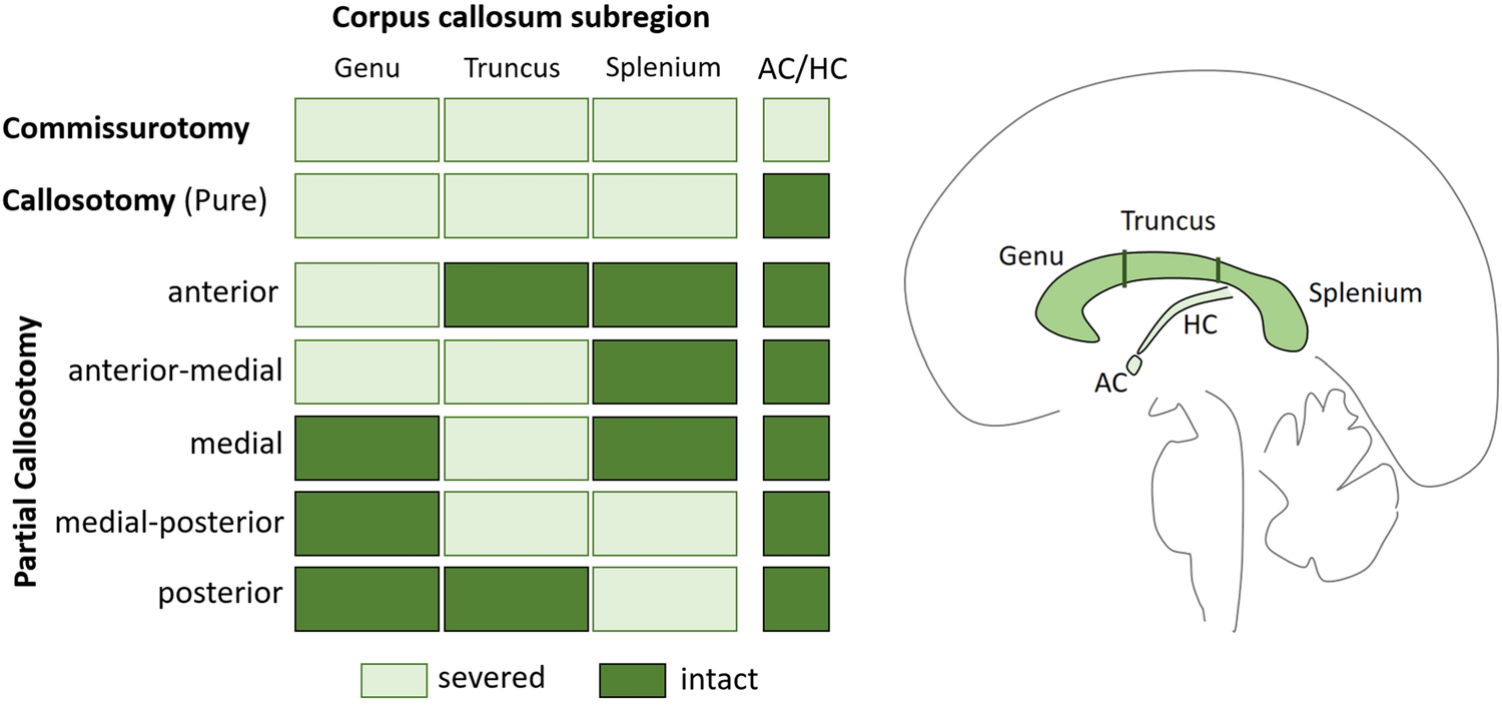
Overview of the categories used to classify the callosotomy patients found in the literature based on the spatial extent of the surgery. Light boxes indicate that the respective subregion was sectioned, while dark boxes indicate the region to be intact. The definition of anatomical subregions follows the division of the corpus callosum into thirds relative to the anterior–posterior extent of the structure as illustrated on the right. AC and HC refer to anterior and hippocampal commissure, respectively

Cases were classified according to the extent of the callosal surgery into three main categories: commissurotomy (complete callosotomy and additional section of anterior and hippocampal commissure), “pure” complete callosotomy, and partial callosotomy. Patients with partial callosotomy were further classified based on which callosal region was affected by assuming a subdivision of the corpus callosum as “thirds” (analogous to the most basic geometrical callosal subdivision introduced by Witelson, 1989) as illustrated in Fig. 3. This approach is only an approximation as the surgical interventions did not follow any clear subdivision scheme as implied by using thirds. The classification was based on the description provided in the original publication, or made by referring to additional articles that provide a general description or MR images the included cases (Bogen et al., 1988; P. M. Corballis et al., 2001; Fabri et al., 2017; Fabri et al., 1999; Gazzaniga et al., 1985; Pallini et al., 1995).

### Data Extraction: Dependent Variable

For each identified patient CUD/bRG values were extracted as well as (where available) mean response times for the various conditions of the paradigm. Likewise, group means for the control groups were extracted where available. If data from one or more experiments were available per publication, data were extracted by experiment and condition (e.g., not as aggregate across conditions). An annotated overview of the data extraction can be found in a pdf document located in the accompanying OSF project (https://doi.org/10.17605/OSF.IO/Y46BV). This document presents for each study the source (e.g., a screenshot of a result table) from which data were extracted as well as conducted calculations by the present author. The data extraction was conducted by a single rater but was validated by recalculating the CUD/bRG from extracted mean response times of crossed, uncrossed, and bilateral conditions and comparing the result with the reported value. Such identified deviations were cross-checked and corrected.

The most basic interhemispheric version of the redundant-target paradigm consists of six conditions, resulting from combining the factors Response Hand (left vs. right) and the Side of Stimulation (unilateral LVF, unilateral RVF, bilateral). The logic of the bRG demands to relate the response time of the bilateral condition to the response time of one or several unilateral reference conditions. This leaves several options for the calculation of bRG. In the split-brain studies, three different approaches have been used to obtain a reference response times for the unilateral condition. That is, (a) the calculation of an average response time to unilateral LVF and RVF stimulation (average reference, see e.g. Roser & Corballis, 2002; Savazzi & Marzi, 2004), (b) the selection of the fastest response time of the two stimulation conditions (minimum reference, see e.g., Corballis, 1998; Ouimet et al., 2009; Roser & Corballis, 2003), or (c) the selection of the response time to the ipsilateral hemifield considering the responding hand (e.g., LVF for left hand response, RVF for right hand response; i.e., ipsilateral reference, see e.g., Corballis et al., 2005; Iacoboni et al., 2000). Supplementary Section A illustrate that the three reference methods may lead to divergent bRG estimates based on example data taken from Experiment 1 reported in Corballis (1998, reproduced in Table S1). Considering patient LB, the average and minimum reference lead to an bRG estimate of 81.3 and 51.0 ms, respectively. Following the assumptions of the Poffenberger paradigm, the ipsilateral (or uncrossed) should be faster than the contralateral condition, so that minimum and ipsilateral references would yield the same bRG estimate. This is the case for patient LB in Tables S2. However, when the assumption is violated – as it is in patient ME – both methods yield different bRG estimates. Thus, the choice of the reference methods may affect the results and this needs to be considered when integrating the individual patient data across studies. Consequently, analyses distinguish the three reference methods. Henceforth, it will be referred to bRG estimates based on average, minimum, and ipsilateral reference as bRG_avg_, bRG_min_, and bRG_ips_, respectively,

### Risk-of-Bias Analysis

All studies underwent a risk of bias analysis. Case studies of callosotomy patients represent a quasi-experimental design as group membership is not randomly assigned. Accordingly, the risk-of-bias analysis was conducted following the seven domains suggested by Sterne et al. (2016) for non-randomised studies of interventions. A detailed evaluation by study can be found in the Supplement, section B (Table S3).

### Statistical Analysis

A series of single-step meta-analyses based on the individual-participant data (Burke et al., 2017) was conducted using linear-mixed modelling (LMM). Across all analysis the general approach was to control for patient- and experiment-specific variance (e.g., different paradigm parameters) in the data by introducing the dummy-coded factors “Patient” (case initials used consistently across studies) and “Experiment” (experimental condition; numbered across studies) as random-effect intercept in the analysis. As several patients were tested in multiple studies and experiments, these two factors were modelled as (partially) crossed random effects.

The fixed-effect part of each analysis was designed to estimate and test for group differences in accordance with the aims of the present meta-analysis. First, estimates of mean CUD and bRG (i.e., bRG_avg_, bRG_min_, and bRG_ips_) in the various patient samples were obtained using an intercept-only fixed-effect part and using the CUD and bRG measures, respectively, as dependent variables. Such estimated intercepts and their confidence interval were interpreted as estimate of the population effect in the respective sample, and the related significance represent a test against “zero.” To ensure robustness of the LMM estimation, we only performed an LMM analysis when at least 15 observations were available for the respective subsample. Mean CUD/bRG of the control samples were calculated as weighted means, using the number of observations as weights, and tested against zero.

Secondly, the differences between patient groups in CUD was tested introducing a Group factor into the fixed-effect section, contrasting patients with commissurotomy, (pure) complete callosotomy, and partial callosotomy (i.e., patients with anterior-medial partial section; other patients were excluded as the number of observations was too small). For bRG measures as dependent variable, the Group data contrasted patients with any complete callosotomy (commissurotomy and pure complete callosotomy) and partial (anterior-medial) callosotomy, as the numbers of cases did not allow for further subdivision of the complete callosotomy sample. Again, the LMM estimation was only performed when at least 15 observations were available for the respective subsample/parameter.

Thirdly, the difference between the patient groups and healthy control samples in CUD and bRG values were set-up using an intercept-only fixed-effect part, but here the dependent variable was a deviation score, that is, the difference between a patient and his/her control sample’s mean value (referred to as ΔCUD and ΔbRG measures, respectively). This approach was necessary as the control-sample data typically were presented in aggregated form (i.e., preventing including individual control data in the analysis) and without measures of dispersion (preventing expressing the patients’ deviation from controls as z-value) in the literature.

Fourthly, the response time measures for the uncrossed and uncrossed unilateral condition of the Poffenberger paradigm as well as the unilateral and bilateral condition of bilateral redundant paradigms were analyzed as LMM, again using the difference between patients and controls (ΔRT) as dependent variable. That is, a Group factor was introduced in the fixed-effect part to test for differences between groups, and models with intercept-only fixed-effect part were used to obtain estimates of mean ΔRT and their deviation from zero.

Finally, the association of CUD and bRG in patient by using CUD as fixed-effect predictor and bRG measures as dependent variable. This analysis was conducted for all three reference versions of the bRG measure (i.e., bRG_avg_, bRG_min_, and bRG_ips_).

All analyses were performed in R (version 4.1.0.). LMM analyses were conducted using the *lme4* package (version 1.1–27; Bates et al., 2015). The 95% confidence intervals (*CI*_*95%*_) for the estimated fixed -model parameters were calculated (using the Wald method) and a t-test against “zero” was conducted based on degrees of freedom obtained using the Satterthwaite's method as implemented in the package *lmerTest* (version 3.1–3; Kuznetsova et al., 2017). Effect sizes for pair-wise group differences or deviation against zero were calculated from the LMM by dividing the difference (i.e. the beta of the effect) by the total variance (as standard deviation) obtained from the model as suggested by (Westfall et al., 2014). While this approach yields an approximation to Cohen’s *d*, it is not identical, and hence is referred to *d*_*m*_. Ordinary Cohen’s *d* calculations were employed for the control-group analyses. Where this calculation method could not be applied (e.g., in case of multiple levels on the same factor), the marginal effect size (*ez*_*m*_*;* the variance explained by the fixed factors alone) was calculated (using routines from the *MuMIn* package, version 1.43.17; Barton, 2020). Weighted mean calculations and weighted testing for the control samples was done with package *weights* (version 1.0.4; Pasek, 2020).

## Results

### Poffenberger Paradigm

In total CUDs of 119 observations were identified of which three were removed as the original studies did not consider the data reliable. The removed data came from two studies (Corballis et al., 2003; Ouimet et al., 2009) and reflecting data collected from the same patient (DDV). The remaining *N*_obs_ = 116 observations (i.e., CUD estimates) originated from *N*_cases_ = 38 unique cases tested *k*_*e*_ = 40 experimental conditions in *k*_*s*_ = 22 different studies. That is, patients were tested with various versions of the Poffenberger paradigm and their data appeared in multiple publications. Of the 38 identified cases, 15 (with 78 observations) had a complete section of the corpus callosum, whereby four of these cases with 39 observations had undergone commissurotomy. There were 23 cases (38 observations) with a partial section of the corpus callosum. Cases with anterior-medial section (i.e., intact splenium) represented most of these cases (18, or 78.3%) and observations (30, or 79.0%).

Sixteen out of the above 22 studies also report data of healthy control participants. That is, mean group-level CUD values were available for 27 experiments/conditions and for a total number of *N*_obs_ = 423 observations.

#### Mean CUD of Patients and Controls

**Fig. 4:**
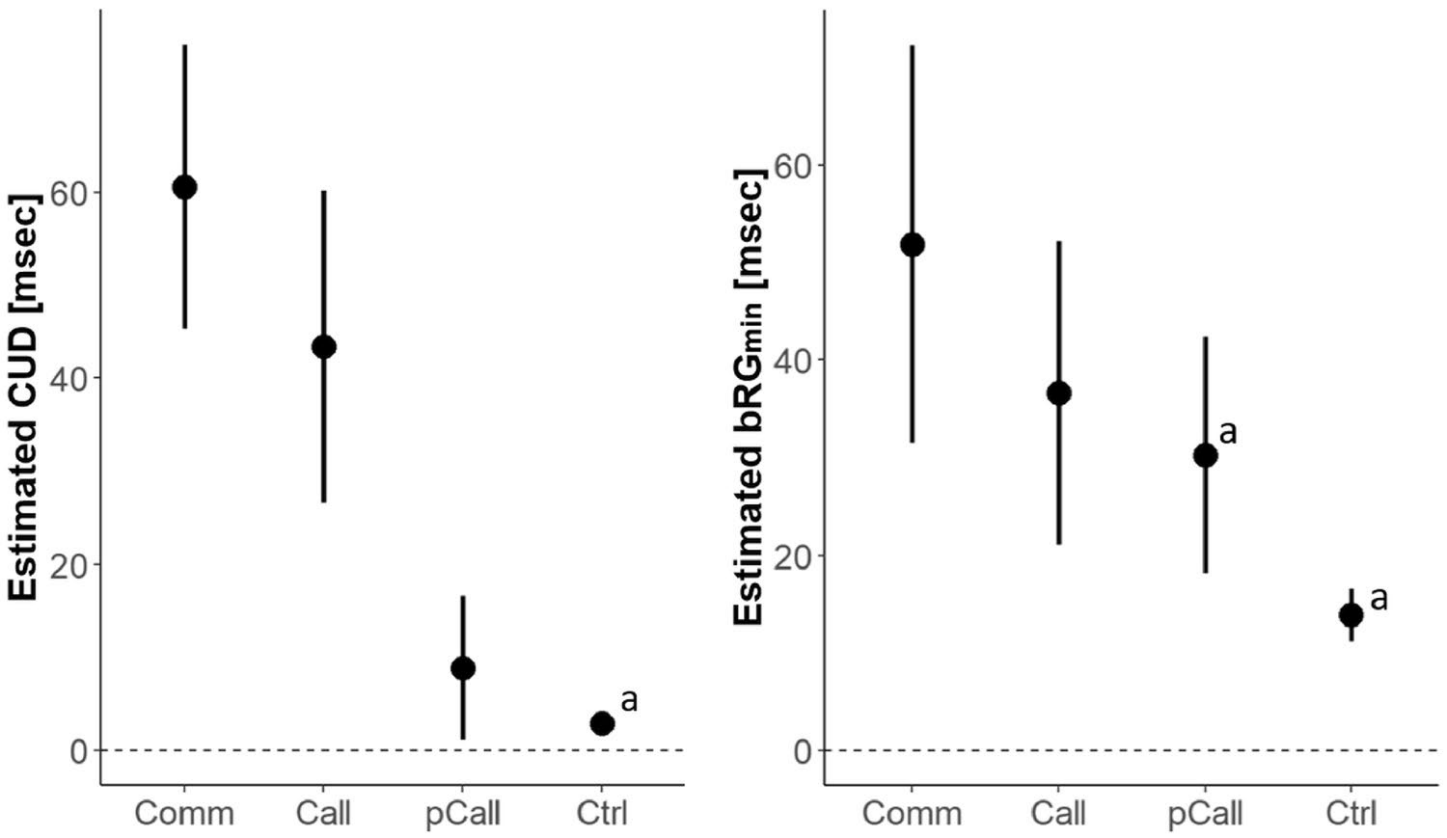
Comparison of the estimated mean and 95% confidence interval of CUD and bRG_min_ in patients with commissurotomy (Comm), with complete (pure) callosotomy (Call), with partial (anterior-medial) callosotomy (pCall), and controls. Estimates marked with an “a” represent (weighted) mean estimates while all other are determined using linear-mixed modelling (for details see text)

The estimated mean and confidence interval of the CUD for all patient groups can be found in Table 2. As can be seen in Fig. 4, the 95%-confidence interval did neither for patients with callosotomy (estimated mean CUD: 43.5 ms) and commissurotomy (60.6 ms) nor patients with anterior-medial partial Sect. (8.8 ms) include zero. For other subtypes of partial section, it was not possible to estimate CUD since the number of observations was too small (see Table 2). In the control sample, the average CUD weighted by sample size was 2.86 ms (*CI*_*95%*_: 2.08; 3.65 ms). A weighted t-test against zero yielded a *t* = 7.15 and was significant (df = 26; *p* < .001, *d* = 1.40).

**Table 2:** Estimates of mean crossed-uncrossed difference (CUD) by patient group

	Sample				CUD			Test statistics			
	N_obs_	N_cases_	k_e_	k_s_	mean^a^	s.e	CI_95%_	*t*-value	*df*	*p*	*d*_m_^b^
*Complete callosotomy*											
Commissurotomy	39	4	18	9	60.6	7.8	45.3; 75.9	7.78	6.5	< 0.001	2.05
Pure callosotomy	39	11	24	13	43.5	8.6	26.7; 60.2	5.07	13.7	< 0.001	1.35
All cases	78	15	36	18	48.9	6.4	36.4; 61.4	7.65	14.6	< 0.001	1.52
*Partial Callosotomy*											
anterior	2	2	1	1	3.2	–^c^	–	–	–	–	–
anterior-medial	29^d^	18	14	9	8.8	3.9	1.1; 16.6	2.24	17.7	0.038	0.52
medial	1	1	1	1	9.6	–	–	–	–	–	–
medial-posterior	4	1	4	2	81.8	–	–	–	–	–	–
posterior	1	1	1	1	6.7	–	–	–	–	–	–
All cases	37	23	15	10	12.9	5.3	2.5; 23.3	2.43	27.9	0.022	0.47

*N*_*obs*_ number of observations, *N*_*cases*_ number of cases, *k*_*e*_ number of experiments/conditions, *k*_*s*_ number of studies

^a^fixed intercept in linear-mixed model (LMM)

^b^effect size as Cohen’ *d* against “zero” estimated from the model variance

^c^not available as number of observations did not allow LMM estimates

^d^value after removing one outlier, with outlier included 15.4 (-2.2; 33.0) ms

Comparing the mean CUD between the three patient groups found a significant main effect of Group (*F*(2, 28.2) = 13.12, *p* < .001; *ez*_*m*_ = 0.34), which reflected that the CUD was significantly larger for the commissurotomy group than the anterior-medial partial section group (estimated differences: *β* = -49.3 ms; *t* = -4.72, *df* = 27.9, *p* < .001) with an effect size of *d*_m_ = -1.85. Commissurotomy and complete callosotomy patients did not differ significantly from each other (*β* = -18.8 ms; *t* = -1.78, *df* = 22.3; *p* = .009 (*d*_m_ = -0.61). The post-hoc pairwise comparison between complete (pure) and partial callosotomy was also significant (*t* = -3.63, *df* = 27.3, *p* = .001; *d*_*m*_ = -1.13). This analysis included data from *N*_obs_ = 107 observations of *N*_case_ = 33 unique cases in *k*_*e*_ = 39 experimental conditions reported in *k*_*s*_ = 21 studies (see Table 2 for subgroup size).

#### Differences Between Patients and Controls (ΔCUD Analysis)

The mean CUD-deviation score (ΔCUD; i.e., individual patient CUD minus its control sample mean CUD) was found to be significant in both commissurotomy and callosotomy patients. That is, the difference for the commissurotomy sample was with *β* = 58.9 ms (*CI*_*95%*_: 43.2; 74.5) and an effect size of *d*_*m*_ = 1.74 significant (*t* = 7.36, *df* = 6.4, *p* < .001). The sample included *N*_obs_ = 37 observations of *N*_cases_ = 4 cases from *k*_*e*_ = 17 experimental conditions (reported in *k*_*s*_ = 8 studies). For the pure callosotomy patients, the intercept was *β* = 34.3 ms (*CI*_*95%*_: 18.2; 50.4) and had an effect size of *d*_*m*_ = 1.32 significant (*t* = 4.17, *df* = 7.7, *p* = .003). Here the sample included *N*_obs_ = 26 observations of *N*_cases_ = 7 cases tested in *k*_*e*_ = 13 experimental conditions (*k*_*s*_ = 8 studies). For patients with an anterior-medial section of the corpus callosum the intercept was *β* = 9.2 ms (*CI*_*95%*_: -1.3; 19.7) and not statistically significant (*t* = 1.72, *df* = 10.7, *p* = .11; *d*_*m*_ = .51). The test included *N*_obs_ = 20 observations of *N*_case_ = 11 patients (*k*_*e*_ = 11, *k*_*s*_ = 6).

#### Response-Time Differences (ΔRT) in Uncrossed and Crossed Conditions

The analysis of the response-time data included *N*_obs_ = 72 observations from *N*_case_ = 21 (*k*_*e*_ = 21, *k*_*s*_ = 11), which represent all data points for which it was possible to calculate a deviation in response time from a control sample (ΔRT). Of these, *N*_obs_ = 30 were from patients with commissurotomy, *N*_obs_ = 18 of patients with pure callosotomy, and *N*_obs_ = 24 from patients with partial callosotomy.

The mean ΔRT of the uncrossed condition did not differ between the three patient groups (i.e., commissurotomy, callosotomy, all partial callosotomy), as indicated by a non-significant main effect of Group (*F*(2, 20.4) = 0.11, *p* = 0.90, *ez*_*m*_ = 0.01). Using separate analyses per group, mean ΔRT were significantly larger than zero for all three groups: commissurotomy patients had a mean value of *β* = 117.7 ms (*CI*_*95%*_: 57.1; 178.4, *t* = 3.81, *df* = 3.8, *p* = 0.02, *d*_*m*_ = 1.42), patients with pure callosotomy a mean of *β* = 171.0 ms (*CI*_*95%*_: 78.6; 263.3, *t* = 3.63, *df* = 9.8, *p* = 0.005, *d*_*m*_ = 1.38), and patients with partial callosotomy a mean of *β* = 138.5 ms (*CI*_*95%*_: 51.3; 225.7, *t* = 3.11, *df* = 11.2, *p* = 0.01, *d*_*m*_ = 0.92).

The mean ΔRT of the crossed condition did not differ significantly between the three patient groups (*F*(2, 20.5) = 0.10, *p* = 0.90, *ez*_*m*_ < 0.01). The estimated mean ΔRT in separate analyses was *β* = 170.6 ms (*CI*_*95%*_: 105.1; 236.1, *t* = 5.11, *df* = 3.9, *p* = 0.008, *d*_*m*_ = 1.87) for the commissurotomy group, *β* = 185.4 ms (*CI*_*95%*_: 127.7; 243.2, *t* = 6.30, *df* = 5.3, *p* = 0.001, *d*_*m*_ = 2.01) for the pure callosotomy group, and *β* = 145.9 (*CI*_*95%*_: 27.8; 264.0, *t* = 2.42, *df* = 11.5, *p* = 0.03, *d*_*m*_ = 0.74) for the partial callosotomy group.

### Bilateral Redundant Target Effect

The literature provided 106 observations of bRG estimates in patients. This includes both bRG estimates provided by the study or calculated based on the response time reported by the studies. Three observations were removed from the analysis. One was removed since the original study reported a very low number of correct trials in one of the conditions and did not consider the data valid (see Corballis et al., 2003; patient DDV in experiment 1). Two were removed as the response was given verbally (see Reuter-Lorenz et al., 1995, experiment 1) in contrast to the motor response mode used by all other studies.

The remaining *N*_obs_ = 103 observations stem from *N*_*cases*_ = 22 unique cases tested in *k*_*e*_ = 24 experimental conditions and reported in *k*_*s*_ = 10 studies. Twelve of these cases had undergone a complete section of the corpus callosum and contributed *N*_obs_ = 79 observations. Three cases (providing *N*_obs_ = 26 observations) had commissurotomy and nine cases (*N*_obs_ = 53) a “pure” callosotomy. All but one of in total *N*_*cases*_ = 10 cases with partial callosotomy had undergone anterior-medial transection. These cases provided *N*_obs_ = 24 observations. The remaining case had medial-posterior section and provided three observations.

As pointed out above, the studies differed with respect to which unilateral reference the bRG was calculated, so that *N*_obs_ = 27 of the 103 observations represent bRG_avg_, *N*_obs_ = 50 bRG_min_, and *N*_obs_ = 26 bRG_ips_. Table 3 shows how many observations were available per patient group when considering the reference used. The number of observations varied substantially, limiting the possibilities for statistical comparisons, as discussed below.

**Table 3:** Estimates of mean bilateral redundancy gain (bRG) by patient group

		Sample				bRG			Test statistics			
Reference	Group	N_obs_	N_cases_	k_e_	k_s_	mean^a^	s.e	*CI*_*95%*_	*t*–value	*df*	*p*	*d*_m_^b^
Average	Pure callosotomy	16	4	13	5	66.6	6.6	53.7; 79.6	10.1	12.33	< 0.001	2.77
	Commissurotomy	8	3	5	3	101	14.5	72.6; 129.4	– ^c^	–	–	–
	*All complete section*	24	7	15	6	82	10.6	61.2; 102.9	7.72	9.36	< 0.001	2.45
	Anterior–medial section	2	2	1	1	22.7	8.4	6.2; 39.0	–	–	–	–
	Medial–posterior	1	1	1	1	119.3	–	–	–	–	–	–
	*All partial section*	3	3	1	1	54.9	32.6	–9.0; 118.7	–	–	–	–
Minimum	Pure callosotomy	25	7	12	6	36.6	7.9	21.1; 52.1	4.625	7.4	0.002	1.35
	Commissurotomy	10	3	6	3	51.9	10.4	31.5; 72.2	–	–	–	–
	*All complete section*	35	10	14	7	42.8	8.0	27.1; 58.4	5.35	15.5	< 0.001	1.41
	Anterior–medial section	14	6	4	2	30.3	6.2	18.1; 42.4	–	–	–	–
	Medial–posterior	1	1	1	1	45.5	–	–	–	–	–	–
	*All partial section*	15	7	4	2	30.8	7.1	16.8; 44.7	4.32	7.6	0.003	1.45
Ipsilateral	Pure callosotomy	12	5	9	5	26.2	4.6	17.2; 35.1	–	–	–	–
	Commissurotomy	8	2	5	3	55.0	15.5	24.6; 85.3	–	–	–	–
	*All complete section*	20	7	11	6	38.9	9.4	20.4; 57.3	4.12	11.0	0.002	1.23
	Anterior–medial section	5	5	2	2	11.6	4.9	1.9; 21.2	–	–	–	–
	Medial–posterior	1	1	1	1	45.5	–	–	–	–	–	–
	*All partial section*	6	6	2	2	17.2	6.9	3.6; 30.8	–	–	–	–

*N*_*obs*_ number of observations, *N*_*cases*_ number of cases, *k*_*e*_ number of experiments/conditions, *k*_*s*_ number of studies

^a^fixed intercept in linear–mixed model (LMM)

^b^Cohen’ *d* against “zero” estimated from the model variance

^c^not available as number of observations did not allow LMM estimates

Eight of the 10 studies also provided data for a control sample. Split by reference method, control sample data concerning bRG_avg_ was available from *k*_*s*_ = 6 studies providing group-mean values for *k*_*e*_ = 15 experiments/conditions, including *N*_obs_ = 156 observations. With regard to bRG_min_, *k*_*s*_ = 5 studies provided 10 means, summarizing data of *N*_obs_ = 146 observations. Finally, bRG_ips_ data of 3 studies with 5 mean values including a total sample of *N*_obs_ = 88 were reported.

#### Mean bRG in Patient and Control Data

The estimated mean and confidence limits bRG of the patient groups can be found in Table 3. The mean group-level bRG only was determined using LMM when at least 15 observations were available. In all these cases, the 95%-confidence interval of the bRG estimate did not include zero. That is, considering the average reference, bRG_avg_ in callosotomy (mean bRG_avg_ = 66.6 ms) and in the combined commissurotomy/callosotomy samples (82.0 ms) were estimated to lie above zero. Considering the minimum method, estimates for the callosotomy (mean bRG_min_ = 36.6 ms), the combined sample (42.8 ms), and for all patient with partially sectioned corpus callosum (30.8 ms) were above. Finally, for the ipsilateral method, an estimated range was above zero for the combined sample of patients with complete section (bRG_ips_ = 38.9 ms). For none of the other groups the sample size was sufficient to calculate a reliable mean effect.

In the control samples, the weighted average bRG_avg_ was 19.1 ms (CI_95%_: 15.9; 22.3 ms) and was significant when tested against zero (weighted *t* = 11.8, *df* = 14, *p* < .001; *d* = 3.14). The average bRG_min_ was 13.9 ms (CI_95%_: 11.2; 16.6 ms) and differed significantly from zero (*t* = 10.1, *df* = 9, *p* < .001; *d* = 3.3). Likewise, mean bRG_ips_ was 15.1 ms (CI_95%_: 9.8; 20.4 ms) and statically significant (*t* = 5.6, *df* = 4, *p* = 0.005; *d* = 2.8).

Given the small number of observations in some subgroups, the group comparison was only performed considering the bRG_min_ and by comparing all patients with complete section to patients with any partial section. The analysis included data from *N*_*obs*_ = 50 patients (35 complete section, 15 partial section) with *N*_*cases*_ = 17 unique cases from *k*_*e*_ = 15 experimental conditions (*k*_*s*_ = 7 studies). The effect of Group was non-significant (*F*(1, 19.9) = 1.25, *p* = 0.27). The effect size for the group difference (*β* = -12.3, reflecting smaller values in the partial-callosotomy group) was *d*_*m*_ = -0.42. The analysis was repeated by removing the one patient with medial-posterior section to obtain a more homogeneous group of patients with selectively anterior-medial partial section. However, this did not change the outcome of the analysis substantially (*F*(1, 19.0) = 1.48, *p* = 0.24; difference: *β* = -13.53, *d*_*m*_ = -0.46).

#### Differences in bRG Between Patients and Controls (ΔbRG)

The test for difference to the control sample was conducted only when 15 or more observations were available per reference method. First, considering bRG_avg_, the mean difference between patients with (pure) complete callosotomy (*N*_*obs*_ = 15, *N*_*cases*_ = 4, *k*_*e*_ = 12 experimental conditions and *k*_*s*_ = 4 studies) and their controls deviated significantly from zero (intercept, i.e., *β* = 45.4 ms; CI_95%_: 29.1; 61.8) with an effect size of *d*_*m*_ = 1.56 (*t* = 5.46, *df* = 11.6, *p* < 0.001). Comparably, for the combined sample of all callosotomized patients (including commissurotomy; *N*_*obs*_ = 24, *N*_*cases*_ = 7, *k*_*e*_ = 14, *k*_*s*_ = 5) the intercept (*β* = 62.5 ms; CI_95%_: 40.6; 84.4) was significant (*t* = 5.60, *df* = 8.9, *p* < 0.001) with an effect size of *d*_*m*_ = 1.82.

Repeating the above analyses for ΔbRG_min_, in patients with “pure” callosotomy (*N*_*obs*_ = 21, *N*_*cases*_ = 7, *k*_*e*_ = 8, *k*_*s*_ = 4) the intercept (*β* = 21.1 ms; CI_95%_: 3.7; 38.5) was found significant (*t* = 2.38, *df* = 8.0, *p* = 0.045) with an effect size of *d*_*m*_ = 0.74. Also considering the combined sample (*N*_*obs*_ = 31, *N*_*cases*_ = 10, *k*_*e*_ = 10, *k*_*s*_ = 5), the intercept (*β* = 28.9 ms; CI_95%_: 10.7; 47.1) was significant (*t* = 3.11, *df* = 12.7, *p* = 0.009) with similar effect size (*d*_*m*_ = 0.89). Additionally, here it was possible to test all patients with partial section (*N*_*obs*_ = 15, *N*_*cases*_ = 7, *k*_*e*_ = 4, *k*_*s*_ = 2), for which the intercept (*β* = 18.1 ms; CI_95%_: 4.1; 32.1) was found significant (*t* = 2.53, *df* = 7.6, *p* = 0.037, *d*_*m*_ = 0.85). For matter of completeness, we repeated this analysis after removing the one patient with medial-posterior section to obtain a sample consisting exclusively of anterior-medial sectioned patients. This rendered the intercept (*β* = 16.3 ms) non-significant (*t* = 2.08, *df* = 6.4, *p* = 0.08, *d*_*m*_ = 0.75), although the confidence interval did not include zero (CI_95%_: 0.9; 31.7).

#### Response-Time Differences (ΔRT) in Bilateral and Unilateral Conditions

The analysis of the response-time data included *N*_obs_ = 47 observations from *N*_case_ = 15 (*k*_*e*_ = 18, *k*_*s*_ = 6), which represent all data points for which it was possible to calculate a deviation in response time from a control sample (ΔRT). Of these, *N*_obs_ = 6 were from patients with commissurotomy, *N*_obs_ = 26 of patients with pure callosotomy, and *N*_obs_ = 15 from patients with partial callosotomy. Given the small number of observations in the commissurotomy group, the group comparison was set-up to contrast all complete with all partial callosotomy patients.

The mean ΔRT of the bilateral condition did not differ between the two patient groups (*F*(1, 17.5) = 0.51, *p* = .48, *ez*_*m*_ = 0.03). Using separate analyses per group, mean ΔRT were found to be significantly larger than zero for both groups: callosotomy patients had an estimated mean ΔRT of 70.7 ms (*CI*_*95%*_: 12.8; 128.6, *t* = 2.39, *df* = 9.4, *p* = .039, *d*_*m*_ = 0.79), and patients with partial callosotomy a mean of 72.0 ms (*CI*_*95%*_: 38.3; 105.6, *t* = 4.19, *df* = 7.7, *p* = .003, *d*_*m*_ = 1.59). Also considering the unilateral condition no group difference was found (*F*(1, 15.0) = 1.22, *p* = .29, *ez*_*m*_ = 0.06). Separate analyses per group yielded an estimated mean ΔRT of 142.8 ms (*CI*_*95%*_: 88.0; 197.6, *t* = 5.10, *df* = 7.9, *p* < .001, *d*_*m*_ = 1.72) for the complete callosotomy group and of 100.6 ms (*CI*_*95%*_: 50.8; 150.3, *t* = 3.96, *df* = 6.8, *p* = .006, *d*_*m*_ = 1.47) for the partial callosotomy group.

### Relationship Between CUD and bRG Indices

**Fig. 5:**
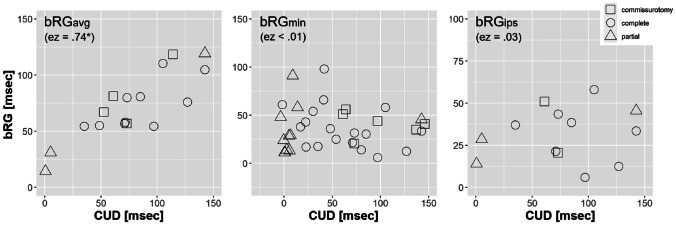
Association of CUD and bRG measures as function of the used unilateral-reference method when calculating bRG. The prediction of bRG from CUD reached statistically significance only for the bRGavg estimate as indicated by the “*”. The different markers indicate patients with commissurotomy (□), complete callosotomy (○), and (any) partial callosotomy (△). The effect size (ez) of the prediction is given as explained variance

The analysis was conducted under consideration of the reference used for calculation of bRG. Considering bRG_avg_, *N*_*obs*_ = 17 observation of *N*_*cases*_ = 10 unique cases (*k*_*e*_ = 8, *k*_*s*_ = 5) having both CUD and bRG_avg_ measures were identified in the literature. As can be seen in Fig. 5, a positive association of the two variables was found. Predicting bRG_avg_ from CUD, the association was statistically significant (*β* = 0.61, *t* = 6.68, *df* = 16.2, *p* = 0.004, *ez* = 0.74). However, CUD did neither significantly predict bRG_min_ (*N*_*obs*_ = 35, *N*_*cases*_ = 17, *k*_*e*_ = 11, *k*_*s*_ = 6; with *β* = 0.05, *t* = 0.61, *df* = 31.3, *p* = 0.55, *ez* = 0.01) nor bRG_ips_ (*N*_*obs*_ = 13, *N*_*cases*_ = 7, *k*_*e*_ = 7, *k*_*s*_ = 4; with *β* = 0.06, *t* = 0.60, *df* = 13, *p* = 0.56, *ez* = 0.03).

## Discussion

Integrating individual-patient data published over the last 50 years, the present series of meta-analyses confirms that both CUD and bRG measures are significantly affected by the complete surgical section of the corpus callosum. While this main finding is in line with the predictions derived from the various case studies summarized in Table 1, the present analyses for the first time overcomes the limited generalizability of individual case reports (Nissen & Wynn, 2014) by providing an estimation of the size of the effect, as well as a statistical evaluation of differences between callosotomy patient groups and of difference between patients and healthy controls.

### Crossed-Uncrossed Differences after Callosotomy

The analysis of the Poffenberger-paradigm data found that the complete section of the corpus callosum (irrespective of as part of a commissurotomy or “pure” callosotomy) results in a CUD estimate ranging between of 36.4 to 61.4 ms (i.e., the 95% confidence interval). This estimated CUD was larger than estimates from healthy controls, as indicated by non-overlapping confidence intervals and significant ΔCUD analyses. A potential difference between patient with commissurotomy and callosotomy could, however, not be confirmed (see also Fig. 4). This result pattern supports the general notion that the corpus callosum is necessary for a fast integration between or coordination of the cerebral hemispheres when performing a Poffenberger paradigm (Marzi, 1999; Miller, 2004; Poffenberger, 1912). The comparison of the CUD after partial and complete callosotomy permits identification of which region of the corpus callosum is most relevant, and the CUD was found to be smaller after partial than (any) complete callosotomy. The confidence intervals of the two patient groups do not overlap and the direct comparison was significant, statistically supporting the observations made in several studies testing both patient groups with the same experiment (Corballis et al., 2003; Ouimet et al., 2010; Tassinari et al., 1994). Importantly, the sample with partial section was dominated by patients who had undergone anterior-medial partial section, so that it appears that an intact posterior corpus callosum (i.e., the splenium, see Fig. 3) is sufficient to substantially alleviate the effect of callosal section on the CUD. The splenium contains axons connecting *inter alia* occipital and parietal cortices (Schmahmann & Pandya, 2006) and is consequently thought to aid vision- or attention-related interhemispheric processing (Bozzali et al., 2012; Tomaiuolo et al., 2010). This would indicate that the integration of visual-perceptual rather than motor processes is the driving factor leading to the CUD since callosal motor pathways are thought to run though more anterior regions (Schmahmann & Pandya, 2006). Examining the few cases which do not fall into the anterior-medial group provides additional support for the special relevance of the splenium. As can be seen in Table 2, a selective anterior (cases BS and GE from Tassinari et al., 1994) or medial section (case WS from Jeeves et al., 2001) is not associated with a substantial CUD increase (all CUD < 10 ms), while a medial-posterior section, affecting the splenium, produces comparatively large CUDs (case MC in Corballis et al., 2003; Savazzi et al., 2007). The seemingly contradictory finding of a small CUD after selective posterior section appears less contradictory knowing that the surgery of this patient apparently spared fibers in the most posterior splenium (case SA, Jeeves et al., 2001), which might allow for a fast inter-hemispheric integration between the visual cortices.

While the above supports the conclusion that a partial callosotomy – especially when sparing the posterior corpus callosum – affects the CUD less severely than a complete section of the corpus callosum, these data do not allow to unequivocally conclude that partial callosotomy has no effect on CUD. That is, the lack of a significant difference of partial anterior-medial callosotomy patients and controls (shown in the meta-analysis of ΔCUD) demands to be interpreted with care since the number of observations is comparatively small. In addition, the potential population-level ΔCUD of up to 19.7 ms (upper 95% confidence limit) cannot be excluded. Likewise, estimates of the range of the “raw” CUD in this patient group – including a slightly larger sample than the ΔCUD analysis – suggests potential values between 1.1 to 16.6 ms, thus ranging well beyond the range of values estimated for healthy controls. Thus, the present analyses cannot be taken to concluded that partial anterior-medial callosotomy does not affect CUD at all, but it supports the notion that it affects the CUD less than a complete section.

It also deserves to be noted that the mean CUD of the control samples was estimated to be between 2.1 to 3.7 ms and, accordingly, was found to be statistically significant. Thus, while only including the controls sample of the callosotomy studies, the present estimate is comparable to findings in a previous, more exhaustive meta-analyses on healthy individuals (Braun, 1992; Marzi et al., 1991). This range also fits well to the estimates obtained in more recent studies (Friedrich et al., 2017; Scally et al., 2018; Westerhausen et al., 2006). From this comparability, one may conclude, that the Poffenberger paradigms utilized in the here reviewed split-brain studies are representative for paradigms typically used for research in healthy individuals. Thus, the conclusions about the paradigm drawn from the present findings likely generalize beyond the use of the paradigm in split-brain research.

### Redundancy Gain after Callosotomy

The meta-analysis of the redundant-target paradigms confirmed the general claim of an increased bRG in split brain patients compared with control samples. The analysis was, however, somewhat limited by studies utilizing different reference methods when calculating bRG, as it subdivided the total sample into subgroups. That is, bRG estimates were obtained by subtracting the response time of the bilateral condition from either the average (Roser & Corballis, 2002), the minimum (e.g., Corballis, 1998; Ouimet et al., 2009), or the ipsilateral response time (Iacoboni et al., 2000) of the unilateral conditions. The data presented in the publications did not always allow to post-hoc recalculate bRG values using another reference method. Consequently, the number of available data points per analysis was smaller than for the Poffenberger paradigm, and it was not possible to distinguish the effects of commissurotomy and (pure) callosotomy. Nevertheless, focusing the discussion on analyses which included 15 or more observation (cf. Table 3), clear evidence for redundancy gain was found in patients with complete section of the corpus callosum. For the largest sample – patients with (any) complete section of the corpus callosum and using the bRG_min_ index – the gain was estimated to be between 27.1 and 58.4 ms (CI_95%_). For the control sample, bRG_min_ was estimated to lie between 11.2 and 16.6 ms so that the none-overlapping confidence intervals with the patient group suggests a significant increase in bRG after complete callosal surgery. This interpretation was confirmed by the deviation analysis showing that ΔbRG_min_ deviated significantly from zero. Thus, the present meta-analysis was able to statistically confirm the general claim of an increased bRG after complete callosotomy as reported in previous case studies (Corballis, 1998; Corballis et al., 2003, 2005; Iacoboni et al., 2000; Ouimet et al., 2009; Pollmann & Zaidel, 1999; Reuter-Lorenz et al., 1995; Roser & Corballis, 2002, 2003; Savazzi & Marzi, 2004).

Patients with partial callosotomy have been less frequently tested with redundant-target paradigms (Corballis et al., 2003; Iacoboni et al., 2000; Ouimet et al., 2009; Reuter-Lorenz et al., 1995) and the reported mean bRG the appear less consistent. For example, while Ouimet et al. (2009; Table 1, p. 687 of original publication) report mean bRG of partial and complete callosotomy that appear roughly comparable, the data from Iacoboni et al. (2000, Fig. 1, p. 762), however, suggest a less pronounced bRG after partial than complete callosotomy. Comparing the two groups in the present meta-analysis did not yield a significant effect. Confidence-interval estimates of the difference between complete and partial anterior-medial callosotomy suggest plausible population values between -35.4 to 8.3 ms. Thus, bRG_min_ population-level difference of up to roughly 35 ms in favor of the partial sectioned group cannot be excluded. In light of the overall mean bRG_min_ of 42.8 in the complete callosotomy group, this potential difference cannot be considered negligible. However, when evaluated in comparison with the control sample, bRG_min_ confidence intervals of patients with partial callosotomy (considering all patients or only patients anterior-medial with partial section) did not overlap with the control samples’ interval, suggesting an increase in redundancy gain. This observation was somewhat supported by the ΔbRG_min_ analysis since the deviation was significant for the sample of all partial-callosotomy patients and present as a trend for the selective anterior-medial callosotomy sample. In both cases, the lower bound of the confidence limit was above zero, suggesting a minimal deviation of 4.1 and 0.9 ms, respectively. Keeping in mind that in extreme case the difference to the control sample are small, the overall pattern suggests that partial callosotomy likely increases the redundancy gain compared to controls. In cannot conclusively determined, however, whether this increase is weaker or comparably strong than the increase after complete section.

### Relationship of CUD and bRG Measures

The analyses of bRG_avg_ and bRG_min_ suggest a significant increase in bRG in patients with complete section compared to controls, whereby the effect size was larger for bRG_avg_. Relatedly, and as can be seen in Table 3, mean estimates for bRG_avg_ in patients with complete section can be considered larger than those of bRG_min_ and bRG_ipsi_ indices. This difference can be attributed to the calculation method, as bRG_avg_ includes the response time of both unilateral condition into the calculation. That is, response times of the condition in which hemifield of stimulation and response hand are crossed and uncrossed are averaged as reference value to determine the gain in the bilateral condition. This also means that the CUD – which here was shown to be increased in patients – is implicitly added into the calculation of bRG_avg_. The other two reference methods, by only using one unilateral condition, avoid this confound. Using the ipsilateral condition (i.e., hemifield and response hand are ipsilateral or uncrossed) or the condition with the fastest response time (i.e., which typically should be the ipsilateral condition) both bRG_min_ and bRG_ips_ are at least mathematically not affected by a slowed CUD. These considerations find support when looking at the empirical association of CUD with the various bRG indices. While CUD can be used to predict bRG_avg_ significantly and with large effect size (70% variance explained), the predictive value for bRG_min_ and bRG_ips_ was substantially lower (< 3% explained variance) and the analysis was not significant.

The association of CUD and bRG indices are, however, also of theoretical importance and sheds light on whether the phenomena rely on the same/partially overlapping interhemispheric-integration processes or are independent of each other (Corballis, 2002). Two competing predictions can be made. First, if CUD and bRG indeed are based on similar callosal processing steps, a longer CUD – reflecting a slower integration between the hemispheres –should affect the bRG negatively. Alternatively, within Miller’s coactivation model (Miller, 2004), it can be predicted that a longer as compared to a shorter CUD results in a slower coactivation of the hemispheres in the unilateral conditions, increasing the bRG. Thus, the here observed strong positive association of CUD and bRG_avg_, taken at face value, would well be in line with the predictions of Miller’s coactivation model. However, as no comparable association was found for bRG_min_ and bRG_ips_ indices, it appears more likely that the found CUD-bRG_avg_ association is due to the discussed mathematical dependency of their calculation rather than any neurophysiological mechanisms. As a note of caution, it must be mentioned that the sample sizes of the bRG_min_ and bRG_ips_ analyses are with 35 and 13 observations, respectively, too small to allow for unambiguous interpretation of the null hypothesis (over alternative small or medium sized effects). However, a second observation of the present meta-analyses that points towards independence of the neuronal mechanisms underlying CUD and bRG is the effect of partial callosotomy on the parameters. As presented above, and keeping the limitations discussed in mind, it appears that partial anterior-medial callosotomy does not substantially increase CUD estimates compared with controls, while it does affect bRG estimates. This dissociation might suggest a critical involvement of the posterior corpus callosum in the neuronal processes reflected in bRG but not in the CUD. Finally, the lack of a substantial association also confirms the findings by Corballis (2002) who examined the relationship in a large sample of healthy individuals. He did not find a significant association (the sample size suffices to exclude population effect of explaining ~ 10% variance or more with a test power of 0.80) and showed by principle-component analysis that the two variables load on different factors. Taken together, it can be concluded that the phenomena bRG and CUD are not strongly dependent on each other, suggesting different underlying neuronal mechanisms.

### Response-Time Measures in both Paradigms

Theoretical considerations make it important not only to analyze the CUD/bRG indices but also to examine the response-time measures of the experimental conditions used to determine these difference scores. This is because callosal-transfer and coactivation models make differential predictions for the unilateral stimulation conditions after callosotomy. The callosal-transfer model assumes functional independence of the two hemispheres when stimulus perception and response initiation are in the same hemisphere (Braun, 1992; Marzi, 1999; Poffenberger, 1912); that is, in uncrossed conditions of both paradigms as well is in the bilateral condition of bRG paradigms. Miller’s graded coactivation model (Miller, 2004) assumes a need for hemispheric interaction (coactivation) in all unilateral stimulation condition (crossed and uncrossed) when initiating a fast response, while in the bilateral condition the coactivation is achieved via direct stimulation, and no callosal effects are predicted (for similar reasoning see Reuter-Lorenz et al., 1995). The present meta-analysis demonstrates the response time of both the uncrossed condition of the Poffenberger paradigm and the bilateral condition of the bRG paradigm to be significantly increased, thus, the findings are at odds with the predictions of both models. Rather the present results can be taken to suggest that the response-time measures of split-brain patients are overall increased irrespective of the experimental condition. This overall slowing might be explained by assuming an unspecific (i.e., not directly related to the callosal location of the surgical intervention) response-time increase of the surgery, as prolonged simple-response times have been revealed comparing pre- and post-surgery performance after split-brain operations (Smith, 1947; patient GV in Tassinari et al., 1994). Alternatively, the general increase may be attributed to response-time slowing existing prior to the surgery and related to the epilepsy condition itself (Breuer et al., 2017; Oyegbile et al., 2018; Tian et al., 2010). Taken together, the present data suggest that the overall elevated mean ΔRT after callosotomy reflects unspecific effects of the surgery or precondition, while the group differences found for CUD/bRG likely reflect effects specific to the callosotomy.

### Limitations

The present meta-analyses on data collected from publications has several limitations. First, the risk-of-bias analysis (see Supplement Section B) identified several potential sources for a systematic bias which are common to almost all studies, as they are inherent to the employed case-study design. All studies attempting the comparison with a control group have the risk of confounding effects, as the control groups are typically healthy individuals rather than other epileptic patients. Thus, the callosotomy patients deviate from their controls in more than just the section of callosal axons. However, for this to systematically bias the present results, it would have to be demonstrated that epilepsy patients show deviant CUD/bRG measures also before the surgery. The evidence for this is scarce. The one callosotomy patient for which pre-surgery data are published – patient GV reported in Tassinari et al. (1994) – does show a CUD that with 8 ms might be considered slightly but not dramatically larger than what is reported in healthy controls. Studies applying Poffenberger/redundant-target paradigms on epilepsy patients outside of the context of callosotomy are, to the best of the author’s knowledge, missing. At the same time, MRI studies of the corpus callosum suggest morphological and microstructural alterations in patients compared with controls (Kim et al., 2008; Weber et al., 2007), which can be seen as indirect evidence that also inter-hemispheric integration might be affected in epilepsy patients irrespective of callosotomy. A second source for bias may arise during the assessment, as the experimenters running the testing are aware of the status of the participant (e.g., patient or control) as typically no “blinding” is reported. Thus, taken together, systematic biases potentially increasing the effect size for the comparisons of patients with controls cannot be excluded but also not verified. Importantly, these potential biases are less likely to affect the comparison between patient groups since both groups are patients, and a differential treatment is less likely.

Second, for several of the conducted meta-analyses the number of available data points was small, reducing the sensitivity of the conducted analyses. This was especially the case for the bRG analyses, as here the various reference methods used required to split up the analyses into smaller subsamples. It would have been ideal to obtain more data points, but the availability of data is naturally limited by the publications that can be found and the lack of contemporary studies on the subject. Thus, acknowledging this limitation, the findings were carefully discussed and interpreted with special focus on the confidence intervals rather than significance level were required. Nevertheless, it is the author’s opinion that the conclusions drawn from the present meta-analytic integration go well beyond what could be achieved by a narrative review of the literature alone.

Third, analyses assessing the deviation of the patient groups from the control group would ideally utilize individual-participant data also for the control sample or at least express the deviation by relating the patient data to the distribution of their control samples (e.g., by calculating z-values). However, the control-sample data was typically presented in aggregated form and often without measures of dispersion preventing the use of both approaches. This made it necessary to use simple mean deviation (here termed ΔCUD, ΔbRG, ΔRT), calculated by subtracting the mean value of the control sample from the individual patient’s value. While this approach is not invalid, it ignores variance in the control sample and might lead to an overestimation of the effect size. However, all conclusions from the analyses using ΔCUD and ΔbRG are confirmed by the comparison of the confidence interval of the “raw” indices between patients and controls. Thus, it appears unlikely the use of simple mean deviations has biased the conclusions of the present study.

Fourth, several studies examining bRG in split-brain patients emphasize that the enhancement is only observed when two redundant stimuli are presented in opposite hemifield/hemispheres but not when they are presented in the same hemifield/hemispheres (Iacoboni et al., 2000; Ouimet et al., 2009; Pollmann & Zaidel, 1999; Reuter-Lorenz et al., 1995). Regarding this unilateral redundancy gain (uRG), the study by Ouimet et al. (2009) is the most interesting, as it includes a larger number of cases (four with complete and four with partial callosotomy) as well as data from a control sample. Across several experimental condition, patients showed a stronger bRG than uRG, whereby the uRG values were in a comparable range to the data from the control sample. While this observation is important, as it further highlights the relevance of between hemifield/hemisphere integration to explain the enhanced bRG, it was not possible to confirm this apparent pattern in a meta-analysis on uRG, as the number of cases was small (even more so when considering the various reference methods).

Finally, the response-time based estimation of CUDs using Poffenberger paradigms has received criticism, as their small magnitude in callosal-intact individuals suggests transfer times that appear physiologically unrealistic (e.g., Saron & Davidson, 1989) or at best represent “*an instance of fastest possible interhemispheric communication* “ (p. 925 in Berlucchi et al., 1995). As alternative, it has been suggested to use the differences in peak latencies of early components of visually evoked potentials (VEP) recorded during the of Poffenberger paradigm to determine transfer time (e.g., Braun et al., 1999; Saron & Davidson, 1989; Scally et al., 2018; Westerhausen et al., 2006). In healthy individuals, this method yields estimates of transfer time which are longer than CUD based estimates (between roughly 10 and 20 ms, instead of 3 or 4 ms) and which have proven more reliable (Friedrich et al., 2017). The few studies that studied split-brain patients (Brown et al., 1998, 1999), interestingly, found contralateral but no ipsilateral VEP after complete commissurotomy (patients LB and NG), supporting the notion that an intact corpus callosum is prerequisite to establish VEP ipsilateral to the stimulated visual field. Given the lack of the ipsilateral VEP, these studies were not able to calculate an interhemispheric transfer time after callosotomy. This observation supports the interpretation that CUD measures determined in split-brain patients likely do reflect subcortical integration that is unrelated to processing in the visual cerebral cortices. However, this observation does not affect the conclusion that here confirmed increased CUD in callosotomy patients as compared with controls, can be attributed to the lack of callosal connections.

## Conclusion

The present meta-analyses are the first to systematically and statistically integrate the findings of case or small-N studies published over five decades to better understand the performance of callosotomy patients in Poffenberger and redundant-target paradigms. The results support the general conclusion that both paradigms assess integration of information between the two hemispheres via the corpus callosum. This can be interpreted as a confirmation of the construct validity of both classes of paradigms as measures of hemispheric interaction. The findings also support the notion that the nature of the assessed integration process likely differs between the two paradigms, as intercorrelations were negligible and the analysis of patients with partial callosotomy show differential findings. This observation justifies future research, using alternative methods to establish the different neuronal mechanism underlying CUD/bRG effects in healthy individuals.

### Supplementary Information

Below is the link to the electronic supplementary material.

Supplementary file1 (DOCX 55 KB)

Supplementary file2 (DOCX 32 KB)

## Data Availability

The annotated tabulated data, which is the basis of the present meta-analysis, and the used R scripts are available in the accompanying Open Science Foundation project (https://doi.org/10.17605/OSF.IO/Y46BV). Together these documents allow to reproduce the here presented analyses. The OSF project page additionally contains the PRISMA checklist and a detailed documentation of the data extraction by study.

## References

[CR1] Aglioti S, Berlucchi G, Pallini R, Rossi G, Tassinari G (1993). Hemispheric control of unilateral and bilateral responses to lateralized light stimuli after callosotomy and in callosal agenesis. Experimental Brain Research.

[CR2] Aglioti S, Tassinari G, Berlucchi G (1996). Spatial stimulus—resonse compatibility in callosotomy patients and subjects with callosal agenesis. Neuroscience & Biobehavioral Reviews.

[CR3] Banich M (1998). Integration of information between the cerebral hemispheres. Current Directions in Psychological Science.

[CR4] Barton, K. (2020). MuMIn : multi-model inference, R package version 1.43.17. Retrieved from https://CRAN.R-project.org/package=MuMIn

[CR5] Bashore TR (1981). Vocal and manual reaction time estimates of interhemispheric transmission time. Psychological Bulletin.

[CR6] Bates D, Mächler M, Bolker B, Walker S (2015). Fitting linear mixed-effects models using lme4. Journal of Statistical Software.

[CR7] Berlucchi G, Aglioti S, Marzi C, Tassinari G (1995). Corpus callosum and simple visuomotor integration. Neuropsychologia.

[CR8] Bernard, R., Goran, D., Sakai, S., Carr, T., McFarlane, D., Nordell, B., & Potchen, E. (2002). Cortical activation during rhythmic hand movements performed under three types of control: an fMRI study. *Cognitive, Affective, & Behavioral Neuroscience, 2*(3), 271–281. 10.3758/cabn.2.3.271 12775191

[CR9] Bogen JE, Schultz DH, Vogel PJ (1988). Completeness of callosotomy shown by magnetic resonance imaging in the long term. Archives of Neurology.

[CR10] Bozzali M, Mastropasqua C, Cercignani M, Giulietti G, Bonni S, Caltagirone C, Koch G (2012). Microstructural damage of the posterior corpus callosum contributes to the clinical severity of neglect. PLoS ONE.

[CR11] Braun CM (1992). Estimation of interhemispheric dynamics from simple unimanual reaction time to extrafoveal stimuli. Neuropsychology Review.

[CR12] Braun CM, Sapinleduc A, Picard C, Bonnenfant E, Achim A, Daigneault S (1994). Zaidel′ s model of interhemispheric dynamics: Empirical tests, a critical appraisal, and a proposed revision. Brain and Cognition.

[CR13] Braun CM, Achim A, Villeneuve LC (1999). Topography of averaged electrical brain activity relating to interhemispheric dynamics in normal humans: where does the critical relay take place?. International Journal of Psychophysiology.

[CR14] Breuer, L., Grevers, E., Boon, P., Bernas, A., Bergmans, J., Besseling, R., & Vonck, K. (2017). Cognitive deterioration in adult epilepsy: clinical characteristics of “Accelerated Cognitive Ageing”. *Acta Neurologica Scandinavica, 136*(1), 47-53. 10.1111/ane.12700 27790700

[CR15] Brown WS, Bjerke MD, Galbraith GC (1998). Interhemispheric transfer in normals and acallosals: Latency adjusted evoked potential averaging. Cortex.

[CR16] Brown WS, Jeeves MA, Dietrich R, Burnison DS (1999). Bilateral field advantage and evoked potential interhemispheric transmission in commissurotomy and callosal agenesis. Neuropsychologia.

[CR17] Burke DL, Ensor J, Riley RD (2017). Meta-analysis using individual participant data: One-stage and two-stage approaches, and why they may differ. Statistics in Medicine.

[CR18] Chechlacz M, Humphreys GW, Sotiropoulos SN, Kennard C, Cazzoli D (2015). Structural organization of the corpus callosum predicts attentional shifts after continuous theta burst stimulation. Journal of Neuroscience.

[CR19] Clarke JM, Zaidel E (1989). Simple reaction times to lateralized light flashes: Varieties of interhemispheric communication routes. Brain.

[CR20] Corballis MC (1998). Interhemispheric neural summation in the absence of the corpus callosum. Brain.

[CR21] Corballis MC (2002). Hemispheric interactions in simple reaction time. Neuropsychologia.

[CR22] Corballis PM, Inati S, Funnell MG, Grafton ST, Gazzaniga MS (2001). MRI assessment of spared fibers following callosotomy: A second look. Neurology.

[CR23] Corballis MC, Hamm JP, Barnett KJ, Corballis PM (2002). Paradoxical interhemispheric summation in the split brain. Journal of Cognitive Neuroscience.

[CR24] Corballis MC, Corballis PM, Fabri M (2003). Redundancy gain in simple reaction time following partial and complete callosotomy. Neuropsychologia.

[CR25] Corballis MC, Corballis PM, Fabri M, Paggi A, Manzoni T (2005). Now you see it, now you don't: Variable hemineglect in a commissurotomized man. Cognitive Brain Research.

[CR26] Corballis MC, Corballis PM, Berlucchi G, Marzi CA (2018). Perceptual unity in the split brain: The role of subcortical connections. Brain.

[CR27] Di Stefano M, Sauerwein HC, Lassonde M (1992). Influence of anatomical factors and spatial compatibility on the stimulus-response relationship in the absence of the corpus callosum. Neuropsychologia.

[CR28] Fabri M, Polonara G, Quattrini A, Salvolini U, Del Pesce M, Manzoni T (1999). Role of the corpus callosum in the somatosensory activation of the ipsilateral cerebral cortex: An fMRI study of callosotomized patients. European Journal of Neuroscience.

[CR29] Fabri, M., Foschi, N., Pierpaoli, C., & Polonara, G. (2017). Split-Brain Human Subjects. In L. J. Rogers & G. Vallortigara (Eds.), *Lateralized Brain Functions. Methods in Human and Non-Human Species.* (pp. 29–78). New York, USA: Humana Press/Springer Nature.

[CR30] Forster B, Corballis MC (1998). Interhemispheric transmission times in the presence and absence of the forebrain commissures: Effects of luminance and equiluminance. Neuropsychologia.

[CR31] Friedrich P, Ocklenburg S, Mochalski L, Schlüter C, Güntürkün O, Genc E (2017). Long-term reliability of the visual EEG Poffenberger paradigm. Behavioural Brain Research.

[CR32] Gazzaniga MS (2000). Cerebral specialization and interhemispheric communication: Does the corpus callosum enable the human condition?. Brain.

[CR33] Gazzaniga MS, Holtzman JD, Deck MD, Lee BC (1985). MRI assessment of human callosal surgery with neuropsychological correlates. Neurology.

[CR34] Genç E, Bergmann J, Singer W, Kohler A (2011). Interhemispheric connections shape subjective experience of bistable motion. Current Biology.

[CR35] Gondan M, Minakata K (2016). A tutorial on testing the race model inequality. Attention, Perception, & Psychophysics.

[CR36] Hershenson M (1962). Reaction time as a measure of intersensory facilitation. Journal of Experimental Psychology.

[CR37] Iacoboni M, Zaidel E (1995). Channels of the corpus callosum Evidence from simple reaction times to lateralized flashes in the normal and the split brain. Brain.

[CR38] Iacoboni M, Ptito A, Weekes NY, Zaidel E (2000). Parallel visuomotor processing in the split brain: Cortico-subcortical interactions. Brain.

[CR39] Ikeda A, Lüders HO, Shibasaki H, Collura TF, Burgess RC, Morris HH, Hamano T (1995). Movement-related potentials associated with bilateral simultaneous and unilateral movements recorded from human supplementary motor area. Electroencephalography and Clinical Neurophysiology.

[CR40] Innocenti, G. M., Schmidt, K., Milleret, C., Fabri, M., Knyazeva, M. G., Battaglia-Mayer, A., & Marzi, C. A. (2022). The functional characterization of callosal connections. *Progress in Neurobiology, 208*, 102186. 10.1016/j.pneurobio.2021.102186 PMC8752969 34780864

[CR41] Jeeves M, Ludwig T, Moes P, Norman W (2001). The stability of compromised interhemispheric processing in callosal dysgenesis and partial commissurotomy. Cortex.

[CR42] Kim H, Piao Z, Liu P, Bingaman W, Diehl B (2008). Secondary white matter degeneration of the corpus callosum in patients with intractable temporal lobe epilepsy: A diffusion tensor imaging study. Epilepsy Research.

[CR43] Kinsbourne M, Zaidel E, Iacoboni M (2003). The Corpus Callosum Equilibrates the Cerebral Hemispheres. The parallel brain: The cognitive neuroscience of the corpus callosum.

[CR44] Kuznetsova A, Brockhoff PB, Christensen RH (2017). lmerTest package: Tests in linear mixed effects models. Journal of Statistical Software.

[CR45] Labache, L., Mazoyer, B., Joliot, M., Crivello, F., Hesling, I., & Tzourio-Mazoyer, N. (2020). Typical and atypical language brain organization based on intrinsic connectivity and multitask functional asymmetries. *Elife*,* 9, *e587229. 10.7554/eLife.58722 10.7554/eLife.58722 PMC7605859 33064079

[CR46] Lassonde M, Ouimet C (2010). The split-brain. Wiley Interdisciplinary Reviews: Cognitive Science.

[CR47] Linnet E, Roser ME (2012). Age-related differences in interhemispheric visuomotor integration measured by the redundant target effect. Psychology and Aging.

[CR48] Lodhia V, Suk CJ, Lim V, Hamm JP, Kirk IJ (2017). Decreased interhemispheric time transfer of visual information in adults with Autistic spectrum disorder using the Poffenberger paradigm. Research in Autism Spectrum Disorders.

[CR49] Marzi C (1999). The Poffenberger paradigm: A first, simple, behavioural tool to study interhemispheric transmission in humans. Brain Research Bulletin.

[CR50] Marzi C, Bisiacchi P, Nicoletti R (1991). Is interhemispheric transfer of visuomotor information asymmetric? Evidence from a Meta-Analysis. Neuropsychologia.

[CR51] Marzi C, Fanini A, Girelli M, Ipata A, Miniussi C, Prior M, Smania N, Thier P, Karnath H-O (1997). Is extinction following parietal damage an interhemispheric disconnection phenomenon?. Experimental Brain Research Series.

[CR52] Marzi, C., Perani, D., Tassinari, G., Colleluori, A., Maravita, A., Miniussi, C., & Fazio, F. (1999). Pathways of interhemispheric transfer in normals and in a split-brain subject A positron emission tomography study. *Experimental Brain Research, 126*(4), 451–458. 10.1007/s002210050752 10422707

[CR53] McKeever W, Gandy R, Lassiter A, Rayport M (1997). Interhemispheric Transfer Time Evidence of Functionally Complete Section of the Splenium in the Enigmatic Callosotomy Case POV or VP. Archives of Clinical Neuropsychology.

[CR54] Meissner TW, Friedrich P, Ocklenburg S, Genç E, Weigelt S (2017). Tracking the functional development of the corpus callosum in children using behavioral and evoked potential interhemispheric transfer times. Developmental Neuropsychology.

[CR55] Miller J (1982). Divided attention: Evidence for coactivation with redundant signals. Cognitive Psychology.

[CR56] Miller J (2004). Exaggerated redundancy gain in the split brain: A hemispheric coactivation account. Cognitive Psychology.

[CR57] Miniussi C, Girelli M, Marzi CA (1998). Neural site of the redundant target effect: Electrophysiological evidence. Journal of Cognitive Neuroscience.

[CR58] Mooshagian E, Iacoboni M, Zaidel E (2009). Spatial attention and interhemispheric visuomotor integration in the absence of the corpus callosum. Neuropsychologia.

[CR59] Nissen T, Wynn R (2014). The clinical case report: A review of its merits and limitations. BMC Research Notes.

[CR60] Ouimet C, Jolicoeur P, Miller J, Ptito A, Lassonde M (2008). Enhanced redundant target effect in callosotomized individuals is not sensory in nature: Evidence from total and partial split-brain individuals. Visual Cognition.

[CR61] Ouimet, C., Jolicoeur, P., Miller, J., Ptito, A., Paggi, A., Foschi, N., & Lassonde, M. (2009). Sensory and motor involvement in the enhanced redundant target effect: a study comparing anterior- and totally split-brain individuals. *Neuropsychologia, 47*(3), 684–692.10.1016/j.neuropsychologia.2008.11.023 10.1016/j.neuropsychologia.2008.11.023 19100276

[CR62] Ouimet, C., Jolicœur, P., Lassonde, M., Ptito, A., Paggi, A., Foschi, N., & Miller, J. (2010). Bimanual crossed–uncrossed difference and asynchrony of normal, anterior-and totally-split-brain individuals. *Neuropsychologia, 48*(13), 3802–3814. 10.1016/j.neuropsychologia.2010.09.003 20833192

[CR63] Oyegbile, T. O., VanMeter, J. W., Motamedi, G., Zecavati, N., Santos, C., Chun, C. L. E., & Hermann, B. (2018). Executive dysfunction is associated with an altered executive control network in pediatric temporal lobe epilepsy. *Epilepsy & Behavior,**86*, 145–152. 10.1016/j.yebeh.2018.04.022 PMC7395827 30001910

[CR64] Page, M. J., Moher, D., Bossuyt, P. M., Boutron, I., Hoffmann, T. C., Mulrow, C. D., & Brennan, S. E. (2021). PRISMA 2020 explanation and elaboration: updated guidance and exemplars for reporting systematic reviews. *bmj, 372*, n160. 10.1136/bmj.n160 PMC8005925 33781993

[CR65] Pallini R, Aglioti S, Tassinari G, Berlucchi G, Colosimo C, Rossi G (1995). Callosotomy for intractable epilepsy from bihemispheric cortical dysplasias. Acta Neurochirurgica.

[CR66] Pasek, J. (2020). Weights: Weighting and Weighted Statistics, version 1.0.2. Retrieved January 1, 2022, from https://CRAN.R-project.org/package=weights

[CR67] Poffenberger AT (1912). Reaction time to retinal stimulation with special reference to the time lost in conduction through nervous centers. Archiv Fur Psychologie.

[CR68] Pollmann S, Zaidel E (1999). Redundancy gains for visual search after complete commissurotomy. Neuropsychology.

[CR69] Reuter-Lorenz PA, Nozawa G, Gazzaniga MS, Hughes HC (1995). Fate of neglected targets: A chronometric analysis of redundant target effects in the bisected brain. Journal of Experimental Psychology: Human Perception and Performance.

[CR70] Roser M, Corballis MC (2002). Interhemispheric neural summation in the split brain with symmetrical and asymmetrical displays. Neuropsychologia.

[CR71] Roser M, Corballis MC (2003). Interhemispheric neural summation in the split brain: Effects of stimulus colour and task. Neuropsychologia.

[CR72] Saron CD, Davidson RJ (1989). Visual evoked potential measures of interhemispheric transfer time in humans. Behavioral Neuroscience.

[CR73] Savazzi S, Marzi CA (2004). The superior colliculus subserves interhemispheric neural summation in both normals and patients with a total section or agenesis of the corpus callosum. Neuropsychologia.

[CR74] Savazzi S, Marzi CA (2008). Does the redundant signal effect occur at an early visual stage?. Experimental Brain Research.

[CR75] Savazzi S, Fabri M, Rubboli G, Paggi A, Tassinari CA, Marzi CA (2007). Interhemispheric transfer following callosotomy in humans: Role of the superior colliculus. Neuropsychologia.

[CR76] Scally B, Burke MR, Bunce D, Delvenne JF (2018). Visual and visuomotor interhemispheric transfer time in older adults. Neurobiology of Aging.

[CR77] Schmahmann JD, Pandya DN (2006). Fiber pathways of the brain.

[CR78] Schmid D, Schenk T (2022). Definition: Redundant target paradigm. Cortex.

[CR79] Schulte T, Pfefferbaum A, Sullivan E (2004). Parallel interhemispheric processing in aging and alcoholism: Relation to corpus callosum size. Neuropsychologia.

[CR80] Sergent J, Myers JJ (1985). Manual, blowing, and verbal simple reactions to lateralized flashes of light in commissurotomized patients. Perception & Psychophysics.

[CR81] Smith KU (1947). Bilateral integrative action of the cerebral cortex in man in verbal association and sensori-motor coordination. Journal of Experimental Psychology.

[CR82] Steinmann S, Leicht G, Andreou C, Polomac N, Mulert C (2017). Auditory verbal hallucinations related to altered long-range synchrony of gamma-band oscillations. Scientific Reports.

[CR83] Steinmann S, Amselberg R, Cheng B, Thomalla G, Engel AK, Leicht G, Mulert C (2018). The role of functional and structural interhemispheric auditory connectivity for language lateralization - A combined EEG and DTI study. Science and Reports.

[CR84] Sterne, J. A., Hernán, M. A., Reeves, B. C., Savović, J., Berkman, N. D., Viswanathan, M., & Boutron, I. (2016). ROBINS-I: a tool for assessing risk of bias in non-randomised studies of interventions. *bmj, 355*. 10.1136/bmj.i4919 PMC5062054 27733354

[CR85] Tassinari G, Aglioti S, Pallini R, Berlucchi G, Rossi G (1994). Interhemispheric integration of simple visuomotor responses in patients with partial callosal defects. Behavioural Brain Research.

[CR86] Thiel, A., Schumacher, B., Wienhard, K., Gairing, S., Kracht, L. W., Wagner, R., & Heiss, W. D. (2006). Direct demonstration of transcallosal disinhibition in language networks. *Journal of Cerebral Blood Flow and Metabolism,**26*(9), 1122–1127. 10.1038/sj.jcbfm.9600350 10.1038/sj.jcbfm.9600350 16757978

[CR87] Tian Y, Dong B, Ma J, Zhou S, Zhou N, Wang K (2010). Attention networks in children with idiopathic generalized epilepsy. Epilepsy & Behavior.

[CR88] Tomaiuolo F, Voci L, Bresci M, Cozza S, Posteraro F, Oliva M, Doricchi F (2010). Selective visual neglect in right brain damaged patients with splenial interhemispheric disconnection. Experimental Brain Research.

[CR89] van der Cruyssen I, Gerrits R, Vingerhoets G (2020). The right visual field advantage for word processing is stronger in older adults. Brain and Language.

[CR90] Volz LJ, Gazzaniga MS (2017). Interaction in isolation: 50 years of insights from split-brain research. Brain.

[CR91] Warlop NP, Achten E, Debruyne J, Vingerhoets G (2008). Diffusion weighted callosal integrity reflects interhemispheric communication efficiency in multiple sclerosis. Neuropsychologia.

[CR92] Weber, B., Luders, E., Faber, J., Richter, S., Quesada, C. M., Urbach, H., & Helmstaedter, C. (2007). Distinct regional atrophy in the corpus callosum of patients with temporal lobe epilepsy. *Brain,**130*(12), 3149–3154. 10.1093/brain/awm186 PMC2770440 17728360

[CR93] Westerhausen R, Karud CMR (2018). Callosotomy affects performance IQ: A meta-analysis of individual participant data. Neuroscience Letters.

[CR94] Westerhausen R, Kreuder F, Woerner W, Huster RJ, Smit CM, Schweiger E, Wittling W (2006). Interhemispheric transfer time and structural properties of the corpus callosum. Neuroscience Letters.

[CR95] Westerhausen R, Gruner R, Specht K, Hugdahl K (2009). Functional relevance of interindividual differences in temporal lobe callosal pathways: A DTI tractography study. Cerebral Cortex.

[CR96] Westerhausen R, Bless J, Kompus K (2015). Behavioral laterality and aging: The free-recall dichotic-listening right-ear advantage increases with age. Developmental Neuropsychology.

[CR97] Westfall J, Kenny DA, Judd CM (2014). Statistical power and optimal design in experiments in which samples of participants respond to samples of stimuli. Journal of Experimental Psychology: General.

[CR98] Whitford, T. J., Kubicki, M., Ghorashi, S., Schneiderman, J. S., Hawley, K. J., McCarley, R. W., & Spencer, K. M. (2011). Predicting inter-hemispheric transfer time from the diffusion properties of the corpus callosum in healthy individuals and schizophrenia patients: A combined ERP and DTI study. *NeuroImage,**54*(3), 2318–2329. 10.1016/j.neuroimage.2010.10.048 10.1016/j.neuroimage.2010.10.048 PMC3006645 20977941

[CR99] Witelson SF (1989). Hand and sex differences in the isthmus and genu of the human corpus callosum. A Postmortem Morphological Study. Brain.

