# Supplementary material for: Interhemispheric Integration after Callosotomy: A Meta-Analysis of Poffenberger and Redundant-Target Paradigms
Source: Neuropsychol Rev. 2022 Dec 9;33(4):872–90. doi: 10.1007/s11065-022-09569-w (PMC10769931; doi:10.1007/s11065-022-09569-w)
Supplement: Supplementary file 1 — Supplementary file1 (DOCX 55 KB) [file 11065_2022_9569_MOESM1_ESM.docx]

**Supplement: *Interhemispheric integration after callosotomy: a meta-analysis of Poffenberger and redundant-target paradigms***

**A. Example calculations for the effect of the unilateral reference on the bilateral redundancy gain (bRG)**

The bRG is determined by subtracting the response time measured after bilateral stimulation form the time measured after unilateral stimulation. However, the literature offers three different methods for calculating the bRG by using a different unilateral reference condition. That is, the unilateral reference is either determined as (a) the average response time of the two unilateral conditions (*average reference*), (b) the fastest response time of the two conditions (*minimum reference*), or (c) the response time of the hand ipsilateral to the stimulated hemifield (*ipsilateral reference*). The resulting bRG measures vary in size as is here illustrated using the data from Experiment 1 reported by Corballis (1998), as shown in Table S1.

| *Table S1*. Response time data (in msec), experiment 1, Corballis (1998) | | | | | | |
| --- | --- | --- | --- | --- | --- | --- |
|  | Unilateral LVF | | Unilateral RVF | | Bilateral | |
| Patient | LH | RH | LH | RH | LH | RH |
| LB | 343 | 549 | 423 | 508 | 303 | 446 |
| JW | 384 | 460 | 445 | 380 | 360 | 361 |
| ME | 309 | 432 | 293 | 247 | 251 | 228 |
| *Note*. LVF/RVF: Left/right visual hemifield; LH/RH: left/right hand response | | | | | | |

The calculated bRG estimates are presented in Table S2.

| *Table S2*. Calculated bRG when using the three reference methods. Shown for left (LH), right hand (RH) responses, and as mean of the two. | | | | | | | | | | | |
| --- | --- | --- | --- | --- | --- | --- | --- | --- | --- | --- | --- |
|  | Average | | |  | Minimum | | |  | Ipsilateral | | |
|  | LH | RH | Mean |  | LH | RH | Mean |  | LH | RH | Mean |
| LB | 80.0 | 82.5 | 81.3 |  | 40.0 | 62.0 | 51.0 |  | 40.0 | 62.0 | 51.0 |
| JW | 54.5 | 59.0 | 56.8 |  | 24.0 | 19.0 | 21.5 |  | 24.0 | 19.0 | 21.5 |
| ME | 50.0 | 111.5 | 80.8 |  | 42.0 | 19.0 | 30.5 |  | 58.0 | 19.0 | 38.5 |
| *Note.* The mean values of LR and RH bRG were used for the meta-analysis | | | | | | | | | | | |

As can be seen in Table S2, bRG differs between the methods, with the average method producing larger values than the two others. Following the assumptions of the Poffenberger paradigm, the ipsilateral (or uncrossed) can be expected to be faster than the contralateral condition. Thus, for patient LB and JW minimum and ipsilateral references yield the same bRG estimate. In patient ME, however, this assumption is violated so that also these two methods yield different estimates.

**B. Risk-of-bias analysis**

The case studies conducted on callosotomy patients represent a quasi-experimental design as the group membership is predefined and not randomly assigned. Thus, the risk-of-bias analysis was conducted following the seven domains suggested by Sterne et al. (2016) for non-randomised studies of interventions. However, the usage of these domains demands some general remarks concerning the applicability of these criteria on the present case studies:

(1) The domain *bias due to confounding* questions whether one prognostic variables for CUD/bRG also predicts the intervention received at baseline. This is difficult to evaluate as little is known about potential determinants of CUD/bRG. However, it also must be acknowledged that healthy controls are typically recruited as control groups and not, for examples, other patients that are indicated to receive a surgery but have not received it yet. Thus, should the condition indicating callosotomy, i.e. intractable epilepsy, have an effect on the outcome variable CUD/bRG, there would be a likelihood of a confounding effect. To evaluate potential biases in this domain, the nature of the control sample was evaluated by study. Where healthy control samples were employed in studies comparing patients and controls, it was judged as “*moderate risk of bias*”, given the lack of knowledge regarding possible effects of patient status on CUD/bRG irrespective of the callosotomy. The comparison of patients with partial and complete callosotomy was considered “*low risk of bias*”, given the same underlying condition.

(2) *Bias in selection of participants into the study*. All studies were checked whether information regarding excluded patients before the surgery were provided. However, for neither of the studies this was the case, and this domain was judged as “*no information*” available for all studies.

(3) *Bias in classification of interventions* is in general unlikely as the patients as cases should be clearly identified for the surgery. This domain was judged as “*low risk of bias*” for all studies.

(4) *Bias due to deviations from intended interventions*. Patients compared with controls will likely receive additional care that goes beyond the callosotomy or are tested more frequently with similar tests (experimental experience), which can be seen as such deviation. Potentially patients are treated differentially after partial than complete callosotomy. However, it also is not clear how and if this additional care influences the outcome measures. As details on the intervention and after care are not reported, all studies comparing patients and controls were judged having a “*moderate risk of bias*” on this domain. Comparisons between patient groups can be considered to have “*low risk of bias*” regarding this domain.

(5) *Bias due to missing data*. Should not be of concern in case studies as the classification and inclusion is based on the fact that the surgery and the testing were successful. However, all publications were examined if any patients were excluded/dropped out, and if the missing data could result in a bias and accordingly evaluated. Where this information was not available, this domain was judged as “*low risk of bias*”.

(6) *Bias in measurement of outcomes*. Firstly, across all studies it can assumed that the outcome assessors are aware of the intervention status, as no blinding is conducted in case studies risking a differential assessment. Secondly, differences between studies exist in the reported details (e.g., some use tables of raw response time values per condition, others only the outcome variables CUD/bRG, or only graphs). Especially when studies used graphs, the data could only be retrieved using data extraction tools. However, this can only introduce a systematic effect if patient and control data are reported differently within the study. Studies with multiple patient/control groups were considered having “*moderate risk of bias*” given the lack of blinding, and all studies were additionally screened for more severe measurement biases.

(7) *Bias in selection of the reported result*. All studies were screened for selective reporting of results that led to exclusion from the meta-analyses. For none of the studies this was the case, and the domain was judged as “*low risk of bias*” across studies.

Table S3 provides an overview of the risk-of-bias across these seven domains.

| *Table S3*. Risk-of-bias assessment per study. | | | | | | | | | |
| --- | --- | --- | --- | --- | --- | --- | --- | --- | --- |
|  |  | Domain | | | | | | | Comment on assessment |
| # | Study | 1 | 2 | 3 | 4 | 5 | 6 | 7 |  |
| 1 | Aglioti et al., 1993^a^ |  |  |  |  |  |  |  |  |
| 2 | Aglioti et al., 1996 |  |  |  |  |  |  |  |  |
| 3 | Clarke & Zaidel, 1989 |  |  |  |  |  |  |  |  |
| 4 | Corballis 1998 |  |  |  |  |  |  |  |  |
| 5 | Corballis et al., 2002 |  |  |  |  |  |  |  |  |
| 6 | Corballis et al., 2003 |  |  |  |  |  |  |  | (ad 5) CUD of patient DDV excluded |
| 7 | Corballis et al., 2005 |  |  |  |  |  |  |  | (ad 5) CUD/bRG of patient DDV excluded Exp.1 |
| 8 | DiStefano et al., 1992 |  |  |  |  |  |  |  |  |
| 9 | Foster & Corballis, 1998 |  |  |  |  |  |  |  |  |
| 10 | Iacoboni & Zaidel, 1995 |  |  |  |  |  |  |  |  |
| 11 | Iacoboni et al., 2000 |  |  |  |  |  |  |  |  |
| 12 | Jeeves et al., 2001 |  |  |  |  |  |  |  |  |
| 13 | Marzi et al., 1999 |  |  |  |  |  |  |  |  |
| 14 | McKeever et al., 1997 |  |  |  |  |  |  |  |  |
| 15 | Mooshagian et al., 2009 |  |  |  |  |  |  |  |  |
| 16 | Ouimet et al., 2009 |  |  |  |  |  |  |  | (ad 5) CUD of patient DDV excluded |
| 17 | Ouimet et al., 2010 |  |  |  |  |  |  |  |  |
| 18 | Pollmann et al., 1999 |  |  |  |  |  |  |  |  |
| 19 | Reuter-Lorenz et al, 1995 |  |  |  |  |  |  |  |  |
| 20 | Roser & Corballis, 2002 |  |  |  |  |  |  |  |  |
| 21 | Roser & Corballis, 2003 |  |  |  |  |  |  |  |  |
| 22 | Savazzi & Marzi, 2004 |  |  |  |  |  |  |  |  |
| 23 | Savazzi et al., 2007 |  |  |  |  |  |  |  |  |
| 24 | Sergent & Myers, 1985 |  |  |  |  |  |  |  |  |
| 25 | Tassinari et al., 1994 |  |  |  |  |  |  |  |  |
| *Notes.* a) for redference please refer to maind ocument; The colour codes are: gray = no information; green = low risk of bias; yellow = medium risk of bis. The domains are numbered following the numbering in the text preceding this table. | | | | | | | | | |

**References**

Corballis, M. C. (1998). Interhemispheric neural summation in the absence of the corpus callosum. *Brain, 121 ( Pt 9)*, 1795-1807. doi:10.1093/brain/121.9.1795

Sterne, J. A., Hernán, M. A., Reeves, B. C., Savović, J., Berkman, N. D., Viswanathan, M., . . . Boutron, I. (2016). ROBINS-I: a tool for assessing risk of bias in non-randomised studies of interventions. *bmj, 355*.
